# Conservation and divergence of cortical cell organization in human and mouse revealed by MERFISH

**DOI:** 10.1126/science.abm1741

**Published:** 2022-06-30

**Authors:** Rongxin Fang, Chenglong Xia, Jennie L. Close, Meng Zhang, Jiang He, Zhengkai Huang, Aaron R. Halpern, Brian Long, Jeremy A. Miller, Ed S. Lein, Xiaowei Zhuang

**Affiliations:** 1 Howard Hughes Medical Institute, Department of Chemistry and Chemical Biology, Department of Physics, Harvard University, Cambridge, MA 02138, USA.; 2 Allen Institute for Brain Science, Seattle, WA 98109, USA.

## Abstract

The human cerebral cortex has tremendous cellular diversity. How different cell types are organized in the human cortex and how cellular organization varies across species remain unclear. Here, we performed spatially resolved single-cell profiling of 4,000 genes using multiplexed error-robust FISH (MERFISH), identified >100 transcriptionally distinct cell populations, and generated a molecularly defined and spatially resolved cell atlas of the human middle and superior temporal gyrus. We further explored cell-cell interactions arising from soma contact or proximity in a cell-type-specific manner. Comparison with the mouse cortex showed conservation in the laminar organization of cells and differences in somatic interactions across species. Notably, our data revealed human-specific cell-cell proximity patterns and showed a markedly increased enrichment for interactions between neurons and non-neuronal cells in the human cortex.

The human cerebral cortex comprises billions of cells of distinct types (1). The spatial organizations and interactions of these cells play a critical role in shaping and maintaining various brain functions (2). For instance, interactions between neuronal and non-neuronal cells are essential for axonal conduction, synaptic transmission, and tissue homeostasis, and are required for normal functioning of the brain (3, 4). Disruption of such cell-cell interactions contributes to various neurological disorders, such as autism (5), schizophrenia (6), and Alzheimer’s diseases (7). Yet, we only have a limited understanding of the organizations and interactions of different cell types in the human cortex.

Recent single-cell RNA sequencing (scRNA-seq) analysis has revealed a diversity of transcriptionally distinct cell populations in the human middle temporal gyrus (MTG) (8). Combining scRNA-seq with microdissection (8), and more recently *in situ* sequencing targeting 120 genes (9), have revealed the laminar organization of these transcriptionally defined neuronal cell types, in particular the excitatory neurons, in the human MTG. These studies, however, did not provide the spatial relationship between cell types at high resolution and a systematic characterization of cell-cell interactions among this high diversity of cell types is still lacking. Single-cell transcriptomics and epigenomics analyses have also provided rich insights into the evolution of cellular diversity and molecular signatures of cell types in mouse, marmoset and human cortex (8, 10, 11), but how the spatial relationship and interactions between different cell types vary across species remains largely unclear.

## Single-cell transcriptome imaging of the human cortex

**Fig. 1.**
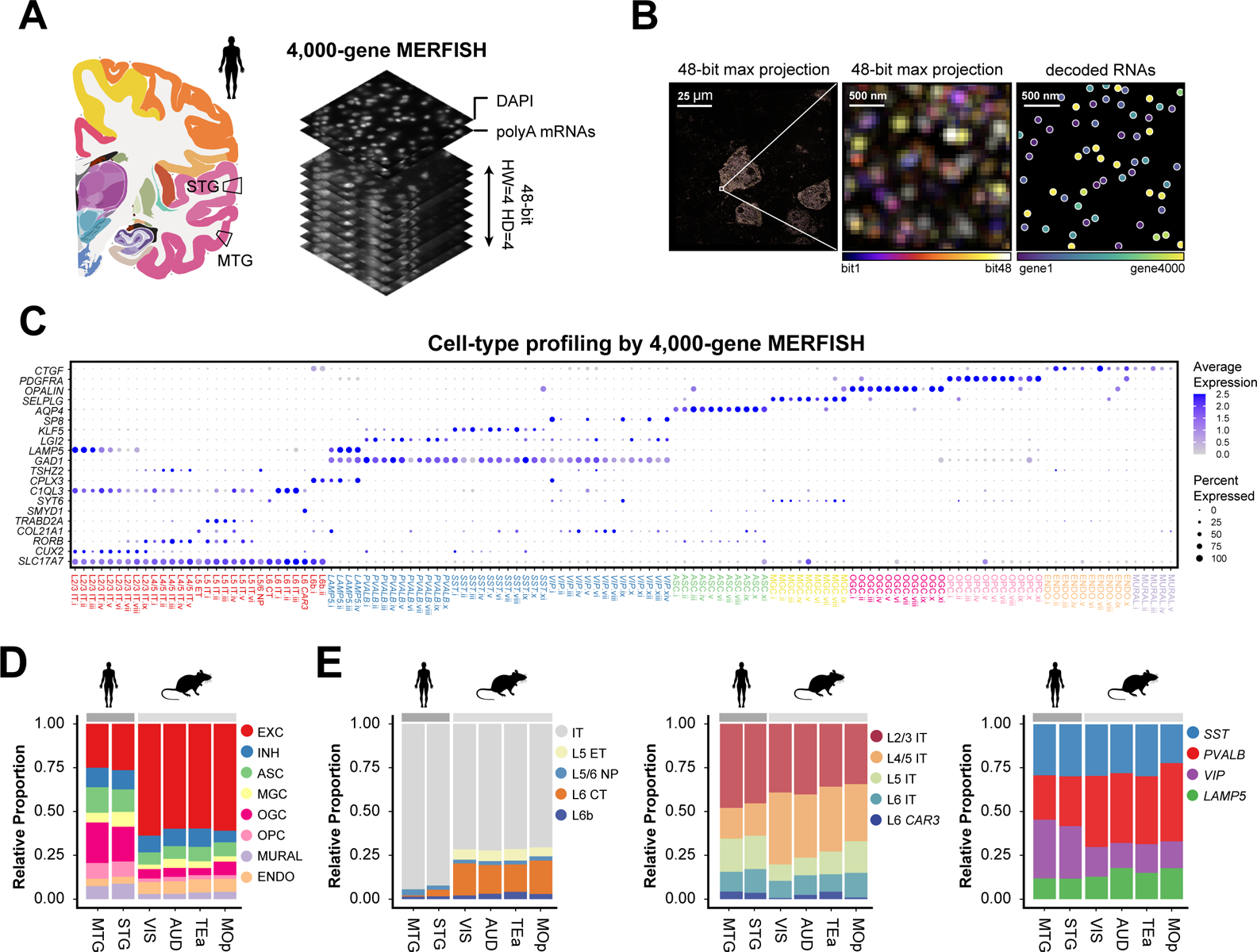
Spatially resolved single-cell transcriptome profiling of the human cortex by MERFISH. (**A**) Schematic of 4000-gene MERFISH measurements of the human MTG and STG using a 48-bit error-correcting code. (**B**) Example MERFISH images. Left: MERFISH image of a single field-of-view, with maximum projection across all 48 bits shown. Middle: Zoomed-in image of the boxed region. Right: Decoded RNA molecules of the zoomed-in region. Scale bars indicate the real size of the sample prior to expansion. (**C**) Cell-type classification of the MTG and STG from MERFISH data and the expression of a subset of marker genes. EXC: excitatory neurons; INH: inhibitory neurons; ASC: astrocytes; MGC: microglial cells; OGC: oligodendrocytes; OPC: oligodendrocyte progenitor cells; ENDO: endothelial cells; MURAL: mural cells; IT: intratelencephalic-projecting neurons; ET: extratelencephalic-projecting neurons; NP: near-projecting neurons; CT: cortico-thalamic projecting neurons. The size and color of each dot correspond to the percentage of cells expressing the gene in each cluster and the average normalized expression level, respectively. (**D**) Proportions of excitatory neurons, inhibitory neurons, and major subclasses of non-neuronal cells in human MTG and STG and four mouse cortical regions including MOp, VIS, AUD and TEa. (**E**) Proportion of subclasses of excitatory neurons (left), IT neurons (middle), and inhibitory neurons (right) in human MTG and STG and the four mouse cortical regions.

Single-cell transcriptome imaging allows *in situ* gene expression profiling of individual cells and hence high-resolution spatial mapping of cell-type organization in complex tissues. Here, we demonstrate single-cell transcriptome imaging of the human brain using multiplexed error-robust fluorescence *in situ* hybridization (MERFISH) (12). We performed MERFISH measurements of the human MTG and superior temporal gyrus (STG) from fresh-frozen neurosurgical and postmortem brain samples, targeting 4,000 genes (Fig. 1A). These genes included 764 differentially expressed marker genes in cell clusters derived from single-nucleus SMART-seq data of the MTG (8) and additional expressed genes largely randomly selected to increase the gene coverage. This allowed us to include potential marker genes not identified in the SMART-seq data, and functionally important genes such as ligands and receptors. To overcome the high autofluorescence background in human tissues due to lipofuscin, we photobleached the samples with light-emitting diode arrays (13) prior to MERFISH imaging. We then used expansion microscopy (14) to reduce the molecular crowding associated with imaging a large number of genes (15, 16).

Individual RNA molecules were identified and assigned to segmented cells to determine the single-cell expression profiles (Fig. 1B; fig. S1). We imaged five tissue sections from neurosurgical MTG samples (two male individuals, 36 and 32 years old) and five sections from postmortem STG samples (two male individuals, 29 and 42 years old). MERFISH expression data showed excellent reproducibility between replicates (fig. S2, A and B), high correlation between neurosurgical MTG and postmortem STG samples albeit with a lower total transcript count in the latter likely due to RNA degradation (fig. S2C), and high correlation with bulk RNA sequencing data (fig. S3).

To test whether the molecular crowding associated with imaging 4,000 genes caused substantial reduction in the detection efficiency, we performed MERFISH imaging on 250 of the 764 marker genes (in two expanded tissue sections for detection efficiency assessment and three additional unexpanded sections to increase the number of cells imaged). The detection efficiency of the 4000-gene measurements was on average ~57% of that of the 250-gene measurements on expanded sections, with high correlation between the two measurements (fig. S4).

## Cell-type classification of the human cortex

We identified transcriptionally distinct cell populations using the single-cell expression profiles derived from the 4,000-gene MERFISH data. First-level clustering identified excitatory and inhibitory neurons, as well as major subclasses of non-neuronal cells including microglia, astrocytes, oligodendrocytes, oligodendrocyte progenitor cells (OPCs), endothelial cells and mural cells, as characterized by the marker genes identified by SMART-seq (8) (fig. S5).

We then performed separate clustering analyses of the inhibitory and excitatory neurons from the MERFISH data, which showed excellent correspondence to those independently determined from the SMART-seq data (fig. S6). To combine information from both datasets, we performed integrated analysis of MERFISH and SMART-seq data (fig. S7, A and B). This analysis classified inhibitory neurons into four subclasses (denoted by marker genes *SST*, *VIP*, *PVALB* and *LAMP5*, respectively) and excitatory neurons into nine subclasses (L2/3 IT, L4/5 IT, L5 IT, L6 IT, L6 IT *CAR3*, L5 ET, L5/6 NP, L6 CT, L6b), with most subclasses further sub-divided into multiple clusters (Fig. 1C). Because non-neuronal cells were depleted from the SMART-seq dataset (8), we identified clusters within individual subclasses of non-neuronal cells from the 4,000-gene MERFISH data alone (Fig. 1C and fig. S7C). Altogether, we identified a total of 125 transcriptionally distinct cell populations in the human MTG and STG, including 29 excitatory, 39 inhibitory, 57 non-neuronal clusters (Fig. 1C; fig. S7), revealing not only a high diversity of neurons but also a high diversity of non-neuronal cells in the human cortex. To include the 250-gene data for downstream analysis, we performed supervised classification to predict their cell type labels (at the cluster level for neurons and subclass level for non-neuronal cells) based on annotations from the 4,000-gene data.

## Cell compositions of the human and mouse cortex

Quantitative analysis of the cell composition using the MERFISH data showed that the human MTG and STG (white matter excluded) were composed of 26% excitatory neurons, 11% inhibitory neurons, and 63% non-neuronal cells (Fig. 1D). The excitatory neurons were predominantly IT neurons (~93%), with only a small fraction of non-IT neurons (L6 CT, L5 ET, L5/6 NP and L6b cells) (Fig. 1E, **left**). The IT neurons were sub-divided into 46% L2/3 IT, 18% L4/5 IT, 19% L5 IT, 13% L6 IT and 4% L6 IT *CAR3* cells (Fig. 1E, **middle**). The inhibitory neurons were composed of 13% *LAMP5*, 26% *PVALB*, 30% *SST*, and 31% *VIP* cells (Fig. 1E, **right**).

Next, we compared the cell composition between human and mouse cortices. Human STG contains the auditory cortex, whereas human MTG does not have a counterpart in mouse, with the mouse temporal associated area (TEa) considered the closest ortholog. We thus considered two MERFISH datasets covering several regions of the mouse cortex: i) our recently reported 258-gene MERFISH dataset of the primary motor cortex (MOp) (17); ii) we additionally performed MERFISH experiments on a more posterior part of the mouse cortex, containing the visual cortex (VIS), auditory cortex (AUD), and TEa (fig. S8) using a similar gene panel and experimental protocol as for the MOp (17). Similar cell compositions were observed across these different mouse cortical regions (Fig. 1, D and E).

The human MTG and STG, however, exhibited substantially different cell composition compared to these mouse cortical regions. We observed a lower proportion of excitatory neurons and a higher proportion of glial cells (including astrocytes, oligodendrocytes, OPCs and microglia) in the human cortex (Fig. 1D). The glia-to-neuron ratio was 1.4, consistent with results from various human cortical regions using other cell counting methods (18, 19), and five times higher than the ratio observed in mouse (Fig. 1D) (17, 20). The excitatory-to-inhibitory neuron ratio was 2.3 in human, in line with recent independent measurements (9, 11) and three times lower than the ratio observed in mouse (Fig. 1D) (17, 20).

Among the excitatory neurons, the non-IT neuron proportion dropped from 29% in mouse to 7% in human (Fig. 1E, **left**), consistent with recent observations that L5 ET and L6 CT are less abundant in the primates than in mouse (11). The dominance of IT neurons in human suggests an increased emphasis of intra-cortical communications in human. For inhibitory neurons, we observed a decrease in the proportion of *PVALB* neurons and an increase in the proportion of *VIP* neurons in human compared to mouse (Fig. 1E, **right**). In behaving animals, *VIP* interneurons regulate inhibition of excitatory neurons through inhibition of other interneurons and such disinhibition facilitates modulation of sensory response and network dynamics by behavioral state and learning (21). The observed increase in *VIP* interneuron proportion thus suggests a potential mechanism for the enhanced capability of state-dependent sensory processing and learning-related neuronal dynamics in human.

## Spatial organizations of cells in the human and mouse cortex

**Fig. 2.**
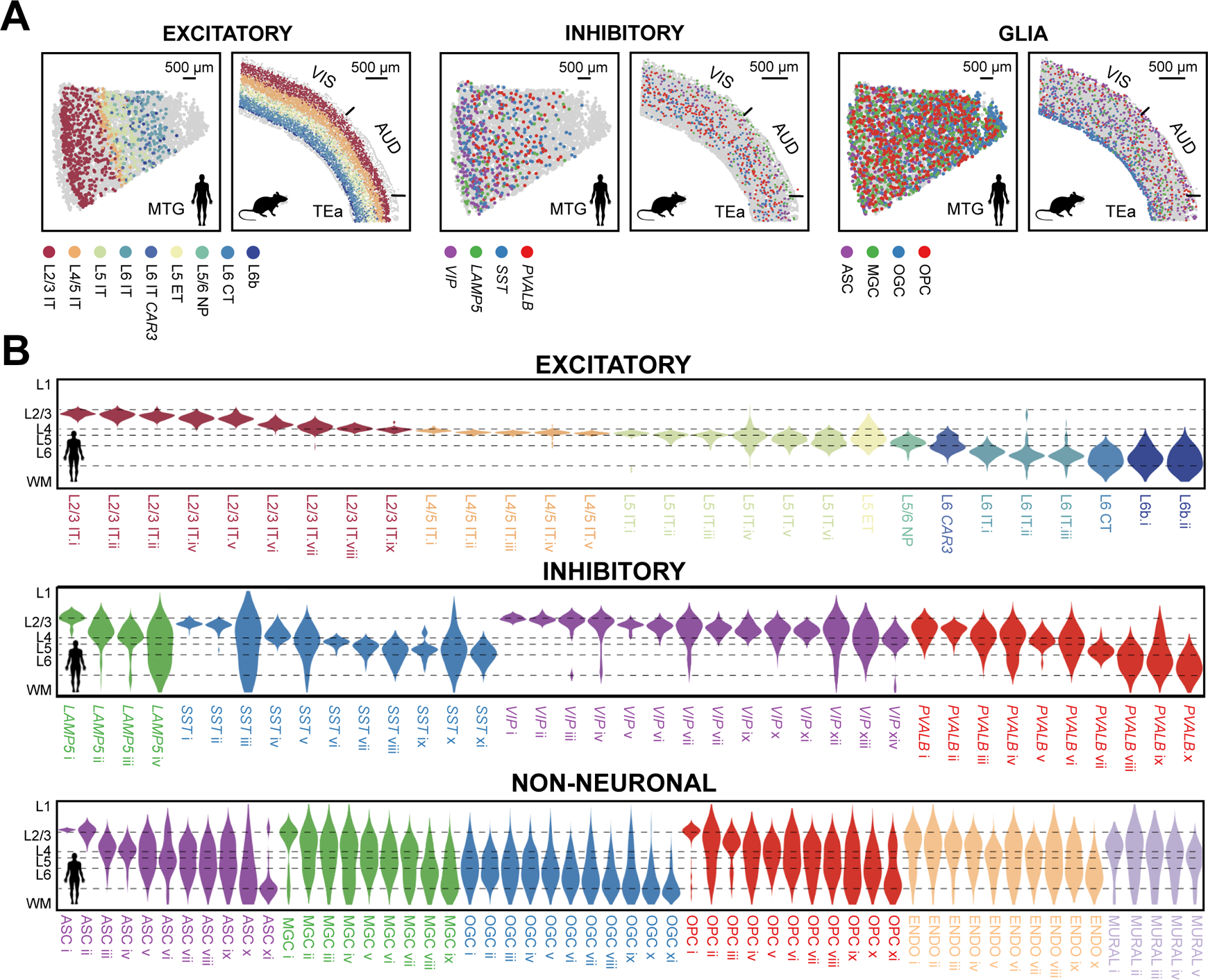
Laminar organization of cell types in the human and mouse cortex. (**A**) Spatial maps of subclasses of excitatory neurons, inhibitory neurons, and glial cells determined by MERFISH in a human MTG slice and a mouse slice containing VIS, AUD and TEa. Indicated subclasses are shown in colors and other cells are in grey. (**B**) Cortical-depth distribution of excitatory (top), inhibitory (middle) and non-neuronal (bottom) clusters in the human MTG. The dashed grey lines mark the approximate layer boundaries. WM: white matter.

*In situ* identification of cell types by MERFISH allowed us to map their spatial organizations. In human, we observed a laminar organization of IT neurons across the cortical depth, whereas other excitatory neurons, including L5 ET, L5/6 NP, L6 CT and L6b, were populated mostly in the deep layers (Fig. 2; figs. S9 and S10), as expected (8, 9, 22). Among inhibitory neurons, *VIP* and *LAMP5* were enriched in upper layers (L1–L3), while *PVALB* and *SST* were more broadly distributed across the layers (Fig. 2; figs. S9 and S10), consistent with pervious observations (8, 9, 22). Notably, at the cluster level, inhibitory neurons also adopted a laminar organization, with many inhibitory clusters primarily restricted to one cortical layer, or even a sub-portion of a layer (Fig. 2B **middle**; fig. S9), enriching and refining the knowledge of layer-restricted inhibitory neuron distributions (8). These spatial organizations of neurons were largely similar to those observed in the mouse cortex (figs. S10 and S11) (17).

**Fig. 3.**
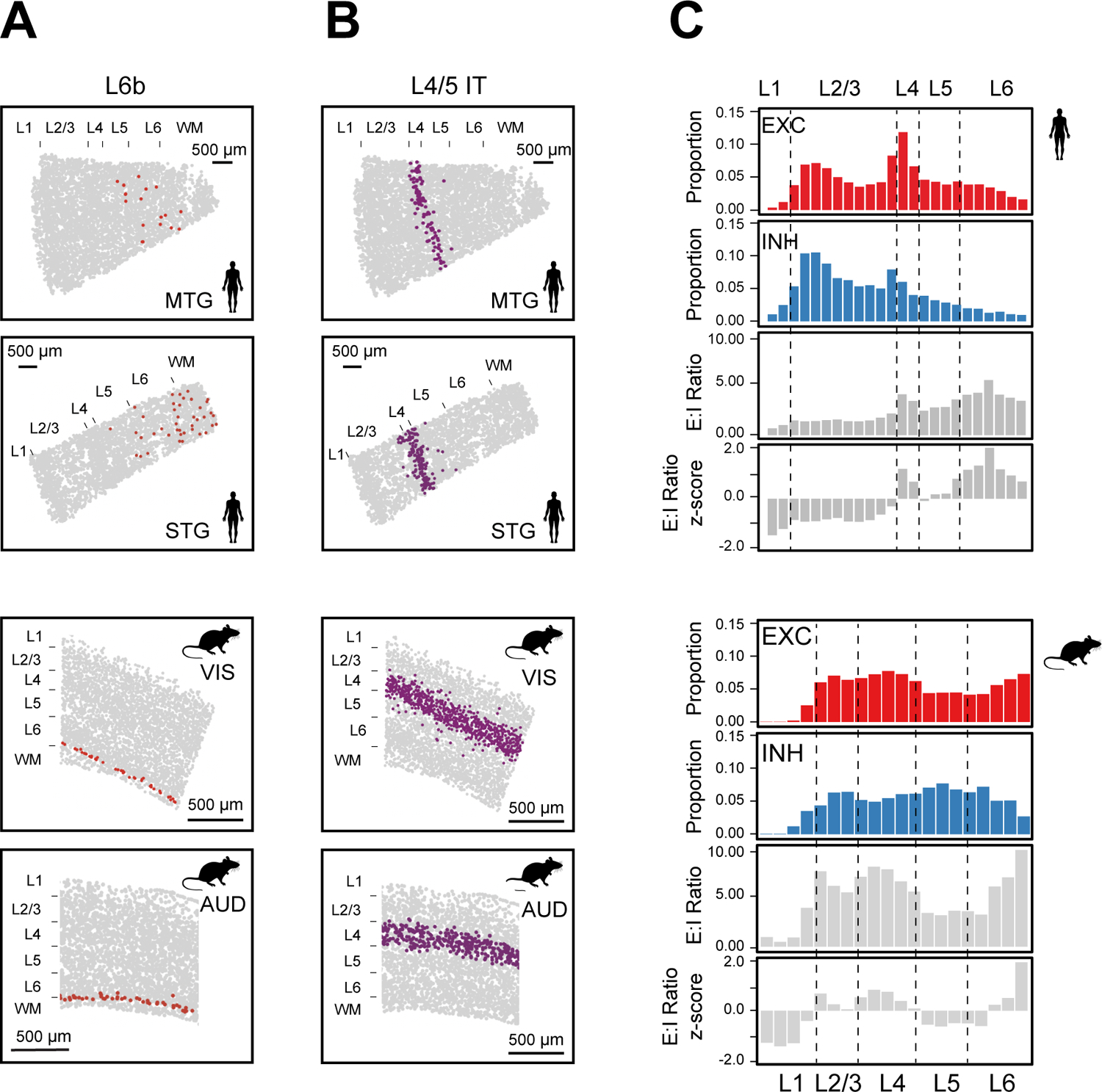
Cortical-depth distributions of L6b, L4/5 IT, and excitatory-to-inhibitory neuronal ratio. (**A-B**) Spatial maps of L6b (**A**) and L4/5 IT (**B**) neurons in a human MTG slice (top), a human STG slice (second), a VIS-containing region (third) and AUD containing region (bottom) in a mouse slice. (**C**) Normalized cortical-depth distributions of excitatory (EXC) and inhibitory (INH) neurons, E:I ratio, and E:I ratio z-score in human (top) and mouse (bottom) cortex. E:I ratio: the ratio between the numbers of excitatory and inhibitory neurons.

Despite the overall conservation of laminar organization, we also found human-mouse differences for some neuronal cell types. For instance, the L6b neurons were broadly dispersed in L6 and extending into L5 and white matter in human MTG and STG, whereas in mouse L6b formed a thin layer at the bottom of L6 (Fig. 3A), consistent with previous findings (22, 23). The L4/5 IT neurons formed a dense and thin layer in the human MTG and STG, giving rise to a substantially higher density of excitatory neurons in L4 (Fig. 3, B and C **top**). In contrast, the density of excitatory neurons in the mouse cortex was more uniform across L2/3 to L6 (Fig. 3C). As L4 is known to vary between different cortical regions, whether this difference is region- or species-specific remains an open question although the several mouse cortical regions that we examined exhibited a similar density profile. We also observed a different cortical-depth dependence for the excitatory-to-inhibitory neuron ratio between human and mouse (Fig. 3C).

Notably, non-neuronal cells also exhibited laminar organization in the human cortex. Oligodendrocytes were enriched in the deeper layers and white matter and depleted in the upper layers (L1–L3) (fig. S10) (9). Although astrocytes, microglia, OPCs, endothelial cells and mural cells were dispersed across all cortical layers at the subclass level (fig. S10), these cell types exhibited a laminar organization at the cluster level (Fig. 2B **bottom**). For example, the ASC i cluster was localized in L1, likely representing interlaminar astrocytes (24), ASC ii was enriched in L1 and L2/3, ASC iii and ASC iv were enriched in L2/3 and L4, ASC v – ix were dispersed across L2–L6, and ASC x and xi were enriched in L6 and white matter (Fig. 2B **bottom**), enriching our understanding of astrocyte diversity and organization (8, 25, 26). Similarly, nearly all non-neuronal subclasses exhibited a gradually evolving cell composition across the cortical depth (Fig. 2B **bottom**).

## Cell-cell interactions in the human and mouse cortex

**Fig. 4.**
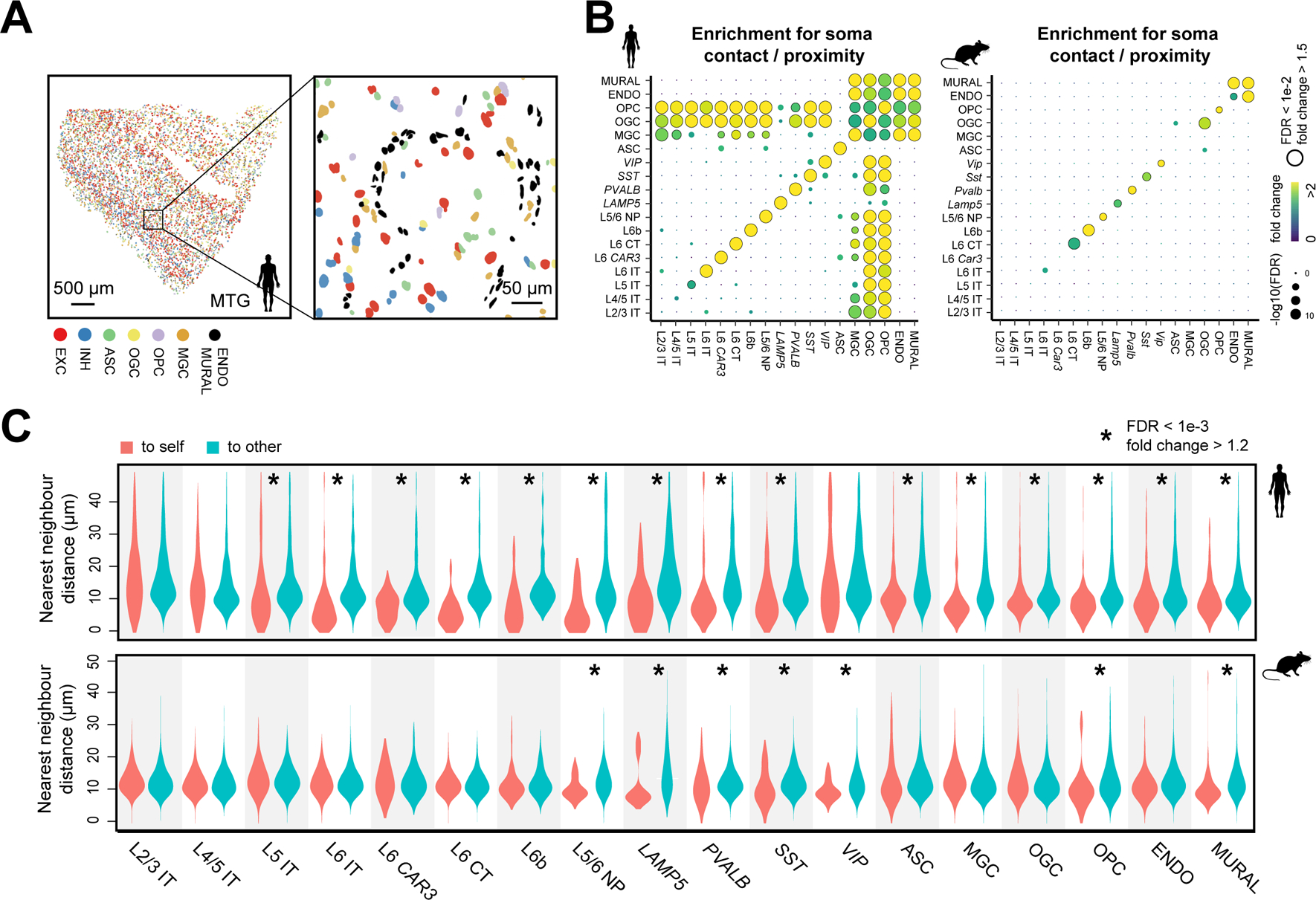
Cell-type-specific cell-cell interactions in the human and mouse cortex . (**A**) Spatial map of excitatory neurons, inhibitory neurons, and six major subclasses of non-neuronal cells in a human MTG slice (left) and a zoomed-in image of the boxed region (right). Colored shapes are cell nuclei segmentations. (**B**) Enrichment map of pairwise soma contact or proximity for subclasses of cells in human (left) and mouse (right) cortex. The color of the dots indicates the fold change between the observed frequency of soma contact or proximity and the average expected frequency from the spatial permutations that disrupt the spatial relationship between neighboring cells (fig. S13). The size of the dots indicates the significance level of the enrichment. FDR: P-value determined with upper-tailed Z-test and adjusted to FDR by the BH procedure. (**C**) Distributions of the nearest-neighbor distances from cells in individual subclasses to cells in the same subclass (“to self”, red) or other subclasses (“to other”, blue) in human (top) and mouse (bottom) cortex. FDR: P-value determined with the Wilcoxon rank-sum one-sided test and adjusted to FDR by the BH procedure.

High-resolution measurements of the spatial relationship between cells by MERFISH allowed us to predict cell-cell interactions arising from somatic contact or paracrine signaling (27, 28), which can be inferred from soma contact or proximity that occurred at a higher frequency than by random chance. Our MERFISH images showed frequent somatic contact or proximity between cells (Fig. 4A; fig. S12, A and B). Interestingly, although the cell density in the human cortex was three times lower than that in mouse (fig. S12C), the median centroid distance between nearest-neighbor cells in human was nearly identical to that in mouse and comparable to the mean soma size (fig. S12, B and D), suggesting that specific mechanisms may exist to maintain or enhance cell-cell interactions in the expanded human cortex. To examine whether these potential cell-cell interactions were cell-type specific, we considered cell types at the subclass level and calculated the frequency at which soma contact or proximity, determined based on centroid distances (fig. S12A), was observed between two subclasses of cells. We then determined whether this frequency was significantly greater than random chance, hence reflecting an enrichment, by comparing the observed frequency with the expected frequencies from random spatial permutations that disrupted the spatial relationship between neighboring cells while preserving the local density of each cell type (figs. S13–S15).

Notably, we observed cell-type specific patterns for soma contact or proximity enrichment in the human cortex that were different from the mouse cortex (Fig. 4B; fig. S16). Similar human-mouse differences were observed when we used segmented cell boundaries instead of distances between cell centroids to determine contacting cell-pairs (fig. S17).

Inhibitory neurons and some deep-layer excitatory neurons in human showed a tendency to form contacting or proximity pairs among cells within the same subclass (Fig. 4B, **left**; fig. S17). These results were further supported by examining the distances from individual neurons to their nearest neighbors in the same or different types (Fig. 4C **top**). This tendency was also observed in mouse but to a lesser degree for some neuronal types (Fig. 4, B and C), consistent with the previous observation that inhibitory neurons in mouse tend to form intra-subtype nearest-neighbor pairs (29). Some non-neuronal cell types also exhibited such tendency for intra-type soma proximity, but with noticeable differences between human and mouse. For example, we observed enrichment for soma contact or proximity among astrocytes in human but not in mouse (Fig. 4B; fig. S17). It has been shown that the processes of neighboring astrocytes intermingle substantially more in human compared to mouse (25, 26, 30). Whether these observations are related to our findings here remains an open question.

**Fig. 5.**
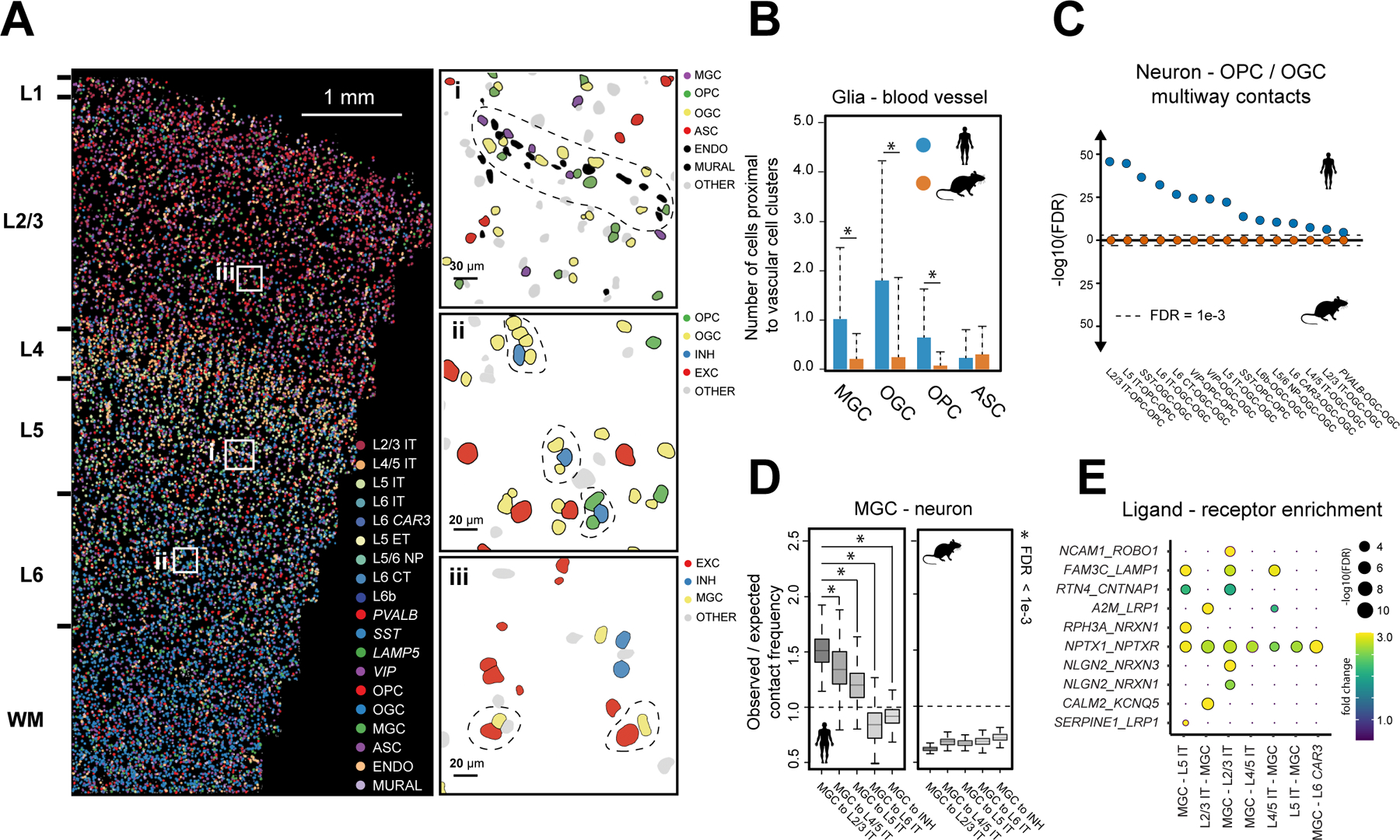
Interactions between glial and vascular cells and between glial cells and neurons in the human and mouse cortex . (**A**) Spatial map of subclasses of cells in a human STG slice. Top right: Zoom-in of boxed region i. A blood vessel with juxtaposed glial cells is marked by dashed line. Middle right: Zoom-in of boxed region ii. Multiway contacts between neurons and oligodendrocytes and/or OPCs are marked by dashed lines. Bottom right: Zoom-in of boxed region iii. Contacting pairs of neurons and microglia are marked by dash lines. Colored and grey shapes are cell nuclei segmentations, (**B**) Average numbers of microglia, oligodendrocytes, OPCs, and astrocytes adjacent to each identified blood vessel in human (blue) and mouse (orange). Error bars are standard deviation (N = 3,415 vascular structures). * FDR < 1e-3 (as determined in Fig. 4B). (**C**) The significance level of multiway contacts between neurons and oligodendrocytes and/or OPCs in human (blue) and mouse (orange) cortex. The significance level was determined by comparing the observed contact frequency with the expected frequencies from spatial permutations as described in fig. S13. FDR: P-values determined with an upper-tailed Z-test and adjusted to FDR by the BH procedure. (**D**) The ratio between observed contact frequency and expected contact frequency (from spatial permutations) between microglia and L2/3 IT, L4/5 IT, L5 IT, L6 IT, and inhibitory neurons in human (left) and mouse (right). In the box plot, midline is the median, box edges are 75^th^ and 25^th^ percentiles, and whiskers indicate 1.5 times the interquartile range. * FDR < 1e-3 (as determined in Fig. 4B). (**E**) Enrichment of ligand-receptor pairs in contacting microglia and IT neurons. The color and size of the dots correspond to the fold change and significance level of the observed ligand-receptor scores over their expected values. FDR as determined in (**C**).

A notable human-mouse difference was observed for glial-vascular interactions. The human, but not mouse, cortex exhibited enrichment for soma contact or proximity between glial and vascular cells (Fig. 4B; fig. S17). MERFISH images showed that the cell bodies of oligodendrocytes and microglia were often clustered around vascular structures formed by endothelial and mural cells (Fig. 5A). These observations are corroborated by a recent electron microscopy study (30), which showed that oligodendrocyte and microglial cell bodies are adjacent to blood vessels whereas astrocytes contact blood vessels primarily with their end feet but not cell bodies. Quantifications of MERFISH images showed that more microglia and oligodendrocytes, but not astrocytes, formed somatic contacts with blood vessels in human than in mouse (Fig. 5B; fig. S18).

The most remarkable cross-species differences on cell-cell interactions were observed between neurons and glial cells, in particular oligodendrocytes and microglia. We observed substantial enrichment for soma contact or proximity between neurons and oligodendrocytes, including both mature oligodendrocytes and OPCs, in human (Fig. 4B; fig. S17). Although somatic contacts between neurons and oligodendrocytes were also observed in mouse and could represent bona fide interactions, the frequency of such events did not significantly exceed that expected from random chance. Moreover, a single neuron often formed contacts with several oligodendrocytes and OPCs in human, whereas such multi-way contacts were not enriched in mouse when compared to random chance (Fig. 5, A and C).

In human, among OPCs, a specific subpopulation exhibited a higher tendency to contact neurons. Our analyses of both MERFISH and SMART-seq data (8) showed that ~50% of the OPCs expressed Glutamate Decarboxylase 1 (*GAD1*), a gene encoding an enzyme that synthesizes GABA, whereas Glutamate Decarboxylase 2 (*GAD2*) and the GABA transporter gene *VGAT* (*SLC32A1*) were not expressed in OPCs (fig. S19A). Compared to *GAD1*-negative OPCs, *GAD1*-positive OPCs exhibited a higher frequency to contact neurons (fig. S19, B and C).

Finally, our data revealed human-mouse differences in microglia-neuron interactions. In the human MTG and STG, microglia frequently juxtaposed with neurons (Fig. 5A), likely representing satellite microglia (31). In addition, these satellite microglia exhibited a greater degree of enrichment for soma contact or proximity with excitatory neurons compared to inhibitory neurons (Fig. 4B, **left**; fig. S17; Fig. 5, A and D). Moreover, among excitatory IT neurons, the tendency to contact microglia decreased with cortical depth (Fig. 5D). In contrast, no significant enrichment in microglia-neuron contact was observed in the mouse cortex (Fig. 4B; fig. S17; Fig. 5D).

Furthermore, we identified ligand–receptor pairs enriched in contacting pairs of microglia and IT neurons from the MERFISH data (Fig. 5E), further validated by single-molecule FISH measurements (fig. S20). Among these, several ligands and receptors are genetically associated with neurodegenerative diseases (Fig. 5E), for instance, Alpha-2-macroglobulin (*A2M*) is genetically associated with Alzheimer’s disease (32), low-density lipoprotein receptor-related protein 1 (*LRP1*) is a master regulator of tau uptake and spread (33), and neurexin (NRXN1/3) is implicated in Autism (34).

## Discussion

Here, we demonstrated 4000-gene MERFISH imaging of human brain tissues. Our MERFISH images enabled *in situ* identification of >100 neuronal and non-neuronal cell populations and comprehensive mapping of the spatial organization of these cells in the human MTG and STG, resulting in a molecularly defined and spatially resolved cell atlas with high granularity. The cell composition in these human cortical regions showed marked differences from that observed in several mouse cortical regions. The spatial organization of cells showed both common and divergent features between human and mouse. Although we cannot exclude the possibility that some of these differences are due to different cortical regions, we consider this less likely because the different mouse cortical regions examined exhibited similar cell-type compositions and organizations, and the same was true for the human MTG and STG.

These high-spatial-resolution cell atlases allowed us to systematically characterize proximity-based somatic interactions in a cell-type specific manner and revealed differences in cell-cell interactions between human and mouse. The differences were particularly striking for interactions between neuronal and non-neuronal cells. We observed substantially increased enrichment for soma contact or proximity between neurons and oligodendrocytes in the human cortex as compared to mouse. Perineuronal oligodendrocytes (35) can provide metabolic support to neurons (36). Hence, the observed increase in contact enrichment between oligodendrocytes and neurons may be a result of evolutionary adaptation to higher energy demands during the firing of individual neurons in the human brain (37). In addition, we observed preferential enrichment for contact or proximity between microglia and excitatory neurons, as compared to inhibitory neurons, in the human cortex, whereas the mouse cortex did not exhibit significant enrichment for such microglia-neuron contact. Satellite microglia can help maintain tissue homeostasis (38) and human genetics evidences suggest that microglia play a protective role that lowers the incidence of some neurodegenerative diseases (39). Our observation may thus represent a functional interaction between microglia and excitatory neurons in human. Interestingly, some ligand-receptor pairs genetically associated with neurodegenerative diseases were enriched in contacting microglia-neuron pairs as compared to non-interacting microglia and neurons, suggesting a possible molecular basis underlying the observed microglia-neuron interactions and a potential connection of these cell-cell interactions to neurodegenerative diseases. It has been suggested that evolution of non-neuronal cells follows a more complex pattern than simply increasing the cell abundance, but additionally involves the diversification of glial cells (40). Our observations of the enhanced enrichment for interactions between neurons and glia in the human cortex further expand upon this view.

## Supplementary Material

Supplementary Materials

Table S1

Table S2

Table S3

Table S4

Table S5

Table S6

## Data Availability

MERFISH data are available at Dryad (41). The SMART-seq data is available at https://portal.brain-map.org/atlases-and-data/rnaseq/human-mtg-smart-seq. All other data are in the main paper or supplement. The MERFISH image acquisition software is available at Zenodo (42). The analysis software is available at Zenodo (43).
