# Supplementary material for: Conservation and divergence of cortical cell organization in human and mouse revealed by MERFISH
Source: Science. Author manuscript; Available in PMC 2022 Jul 8. (PMC9262715; doi:10.1126/science.abm1741)
Supplement: Supplementary Materials [file NIHMS1820958-supplement-Supplementary_Materials.pdf]

Science
AAAS

Supplementary Materials for

Conservation and divergence of cortical cell organization in human and mouse revealed by MERFISH

Rongxin Fang, Chenglong Xia, Jennie L. Close, Meng Zhang, Jiang He, Zhengkai Huang, Aaron R. Halpern, Brian Long, Jeremy A. Miller, Ed S. Lein, Xiaowei Zhuang

Correspondence to: zhuang@chemistry.harvard.edu (X.Z.)

This PDF file includes:

Materials and Methods
Figs. S1 to S20
Captions for Tables S1 to S6

Other Supplementary Materials for this manuscript include the following:

Tables S1 to S6

Materials and Methods

Ethical compliance

Surgical specimens were obtained from local hospitals (Harborview Medical Center, Swedish Medical Center and University of Washington Medical Center) in collaboration with local neurosurgeons. All patients provided informed consent and experimental procedures were approved by hospital institute review boards before commencing the study. De-identified postmortem human brain tissue was collected after obtaining permission from decedent next-of-kin. The Western Institutional Review Board (WIRB) reviewed the use of de-identified postmortem brain tissue for research purposes and determined that, in accordance with federal regulation 45 CFR 46 and associated guidance, the use of and generation of data from de-identified specimens from deceased individuals did not constitute human subjects research requiring institutional review board review. Postmortem tissue collection was performed in accordance with the provisions of the United States Uniform Anatomical Gift Act of 2006 described in the California Health and Safety Code section 7150 (effective 1/1/2008) and other applicable state and federal laws and regulations. Tissue procurement from neurosurgical donors was performed outside of the supervision of the Allen Institute at local hospitals, and tissue was provided to the Allen Institute under the authority of the institutional review board of each participating hospital. A hospital-appointed case coordinator obtained informed consent from donors before surgery. Tissue specimens were de-identified before receipt by Allen Institute personnel. The specimens collected for this study were apparently non-pathological tissues removed during the normal course of surgery to access underlying pathological tissues. Tissue specimens collected were determined to be non-essential for diagnostic purposes by medical staff and would have otherwise been discarded.

Human brain processing

Postmortem human brains were transported on ice and processed as bisected hemispheres by embedding in Cavex Impressional alginate (Cavex Life Cast Normal Aglinate) prepared according to manufacturer's instructions. Each cerebral hemisphere was then cut into 1 cm slabs and individually frozen in an isopentane and dry ice slurry.

Each slab was then vacuum sealed and stored at -80 °C. STG samples were taken by allowing selected slabs to equilibrate to -20 °C for 2 hours. The region of interest was identified and dissected from the slab at -20 °C using a scalpel, vacuum sealed and stored at – 80 °C. Neurosurgical brain tissues, after resection, were bubbled in carbogen and artificial cerebrospinal fluid until vibratome sectioning at 100 µm thickness. Tissue slices were arranged against the flat surface of an embedding mold and covered in Optimum Cutting Temperature Compound (VWR,25608-930). Slices were then flash frozen in a dry ice and ethanol slurry, vacuum sealed, and stored at -80 °C.

Animals

Adult C57BL/6 male mice aged 57-63 days were used in this study. Animals were obtained from Jackson Laboratory (RRID:IMSR_JAX:000664) maintained on a 12 hour: 12-hour light/dark cycle (2pm-2am dark period) with ad libitum access to food and water. Animal care and experiments were carried out in accordance with NIH guidelines and were approved by the Harvard University Institutional Animal Care and Use Committee (IACUC, animal protocol #10-16-3). Mice were euthanized with CO_2, and their brain were quickly harvested and cut into hemispheres and each hemisphere was frozen immediately on dry ice in optimal cutting temperature compound (Tissue-Tek O.C.T.; VWR, 25608-930), and stored at -80℃ until cutting. Frozen brain hemispheres were sectioned at -20℃ on a cryostat (Leica CM3050 S). Slices were removed and discarded until the middle of visual cortex region was reached. We then cut two consecutive 10-µm-thick slices near the middle of visual cortex from each mouse and placed them onto coverslips for imaging. In total two slices were collected for each animal and four slices were imaged.

MERFISH gene selection

To identify transcriptionally distinct cell populations in the human MTG and STG with MERFISH, we designed a panel of 4,000 genes. In details, we first use a combination of two approaches, mutual information (MI) analysis and differentially expressed (DE) gene analysis, to select marker genes based on previously published single nucleus

SMART-seq data in the human MTG (*8*), as described previously (*17*, *44*). In the first approach, we selected the top 300 genes with the greatest MI values for excitatory neuronal clusters and inhibitory neuronal clusters, respectively. In the second approach, we included all DE genes for each cluster when compared to the remaining cells in its respective group, namely excitatory or inhibitory neuron group. This yielded 486 DE genes for excitatory neuronal clusters and 599 DE genes for inhibitory neuronal clusters. We then removed overlapping genes among these 4 groups of MI and DE genes, resulting in a total of 1,233 marker gene candidates. Next, we then added the manually and computationally selected genes provided by the Chan Zuckerberg Initiative SpaceTx Consortium that did not overlap with the 1,233 genes described above, resulting in 1,680 maker gene candidates. To finalize the maker gene list, we removed genes that were either not long enough to construct 60 target regions (each 30-nucleotide (nt) long) without overlap or whose expression levels were outside the range of 0.01 to 500 FPKM, as measured by bulk RNA-seq. This yielded 764 marker genes. For the remaining 3,236 genes, we randomly selected genes that satisfied the following three criteria: 1) the genes were long enough to construct 60 target regions without overlap; 2) the express levels of the genes were within the range of 0.01 to 500 FPKM; 3) the genes did not overlap with 764 cell type marker genes described above.

For MERFISH imaging of the mouse VIS, AUD and TEa, we used a gene panel similar to that previously described for the mouse MOp (*17*). This MOp panel included 242 genes for MERFISH imaging and 16 genes for sequential, two-color FISH imaging following the MERFISH run. Only the 242 genes were imaged here.

<u>Design and construction of the MERFISH encoding probes</u>

For human brain imaging, each RNA species was encoded with a unique binary codeword from a set of 48-bit, Hamming Distance 4 (HD 4), Hamming Weight 4 (HW 4), binary barcodes (**Table S1**). This codebook contains 4,140 unique barcodes and every barcode was separated by a Hamming distance of at least 4 from all other barcodes. Each of 4,000 selected RNA species was randomly assigned a unique binary barcode

from all 4,140 possible 48-bit barcodes. The remaining unassigned 140 barcodes were left as blank controls for misidentification quantification.

For each RNA species, we designed 60 non-overlapping encoding probes to imprint the selected binary codeword onto the corresponding transcript, each encoding probe containing a 30-nt target sequence complementary to one of the 30-nt target regions of the transcript. The target regions were identified using an algorithm as described previously (*45*). In brief, we selected target regions that had a GC fraction between 30% and 70%, a melting temperature TM within the range of 60-80 °C, an isoform-specificity index between 0.75 and 1, a gene-specificity index between 0.75 and 1, and no homology longer than 15 nt to rRNAs or tRNAs. Each of the 48 bits was then assigned a 20-nt three-letter readout sequence. Afterwards, each encoding probe was constructed to contain a unique 30-nt target region and three of the four 20-nt readout sequences corresponding to the four '1' bits assigned to the transcript. The encoding probes additionally contained two priming regions for amplification: a 20-nt primer binding region at the 5' end and the reverse complement of the T7 promoter at the 3' end as previously described (*45*). The encoding probe libraries were purchased from Twist Biosciences and amplified as previously described (*45*). The encoding probe sequences for 4,000 genes are listed in **Table S2**. To test whether the molecular crowding associated with imaging 4,000 genes caused a substantial reduction in the detection efficiency, we also performed MERFISH imaging on 250 genes chosen from the 764 marker genes described above. The codebook for 250-gene is listed in **Table S3** and encoding probe sequences for 250 genes are listed in **Table S4**. Encoding probes for the mouse brain imaging were as described previously (*17*).

Design and construction of the MERFISH readout probes

For human brain imaging, 48 readout probes complementary to the 48 readout sequences were designed, each corresponding to one of the 48 bits in the barcodes. Readout probes were conjugated to one of the dye molecules (Alexa750, Cy5 and Atto565/Cy3B) via a disulfide linkage, as described before (*45*). These readout probes were synthesized and purified by Biosynthesis, Inc., resuspended immediately in Tris-

EDTA (TE) buffer, pH 8 (Thermo Fisher) to a concentration of 100 µM and stored at -20 °C. The readout sequences used in this study are listed in **Table S5**. Readout probes for the mouse brain imaging were as described previously (*17*).

Sample staining

Human tissue slices were allowed to briefly thaw on coverslips at room temperature, and then were fixed by treating with 4% PFA in 1×PBS for 10-15 minutes. We then washed coverslips three times with 1×PBS and stored them in 70% ethanol at 4°C for overnight to permeabilize cell membranes. After permeabilization, tissue slices were exposed under the multi-band light emitting diode arrays for three hours to eliminate the autofluorescence background (*13*). To perform MERFISH encoding probe staining, samples were incubated for 30 min in encoding wash buffer comprising 2× SSC (Ambion), 30% (vol/vol) formamide (Ambion), and 0.1% (vol/vol) murine RNase Inhibitor (New England Biolabs). A drop of 60 µL of 4,000- or 250-gene encoding probes (~1 nM per encoding probe, 60 probes per gene) and acrydite-modified poly(dT) locked nucleic acid (LNA) probes with a unique 20-nt readout sequence (2 µM) in encoding hybridization buffer was added to a parafilm in a 150mm-diameter dish and were covered with tissue-containing coverslips. Encoding hybridization buffer was comprised of encoding wash buffer supplemented with 0.1% (wt/vol) yeast tRNA (Thermo Fisher), 0.1% (vol/vol) murine RNase Inhibitor (New England Biolabs), and 10% (wt/vol) dextran sulfate (Sigma). The samples were then incubated in a cell culture incubator at 37 °C for ~48 hr, followed by washing with encoding wash buffer twice at 47 °C for 30 min. The samples then were incubated for 10 min with a 1:500 dilution of 0.12-µm-diameter light yellow beads (Spherotech, FP-0245-2), which were used as fiducial markers to align images obtained from sequential rounds of hybridization. Mouse brain sample staining was performed as described previously (*17*).

Sample expansion and / or tissue clearing

To expand the human tissue samples labeled with MERFISH encoding probes, we adopted a previously published gel embedding and expansion protocol (*15*, *16*, *46*). Briefly, the samples were first incubated for 10 min in degassed monomer solution

consisting of 2 M NaCl, 7.7% (wt/vol) sodium acrylate (Sigma), 4% (vol/vol) of 19:1 acrylamide/bisacrylamide, 60 mM Tris-HCl pH 8 and 0.2% (vol/vol) TEMED (for buffer exchange). The remaining monomer solution was then kept on ice and further added 0.2% (wt/vol) ammonium persulfate just before casting the expansion gel.

To cast the gel, 25 µL of the ice-cold monomer solution containing 0.2% (wt/vol) ammonium persulfate was added to the surface of a glass plate that had been treated with GelSlick (Lonza). Coverslips containing the encoding-probe-labeled tissue slices were dried quickly with KimWipes (Kimtech) and inverted onto the 25 µL droplet to form a uniform thin layer of gel solution between the coverslip and the glass plate. The sandwiched coverslip and glass plate with gel solution were then transferred to a nitrogen-filled chamber for 2 hours to complete gel polymerization. The coverslip and the glass plate were then separated by using a thin razor blade. The coverslips with expansion gels were transferred to a digestion buffer, which was made of 2% (wt/vol) Sodium dodecyl sulfate (SDS) (Thermo Fisher), 0.5% (vol/vol) Triton X-100 and 1% (vol/vol) Proteinase K (New England Biolabs) in 2× SSC. The samples were digested in digestion buffer overnight in a 37 °C incubator. After digestion, samples were expanded in 1 × SSC buffer supplemented with 0.2% (vol/vol) Proteinase K at room temperature. During expansion, the buffer was changed every 20 minutes for three times.

To stabilize the expanded gel for sequential rounds of readout probe hybridization and imaging, we re-embedded the expansion gel in a nonexpandable polyacrylamide gel. Briefly, expanded polyacrylite gel was buffer exchanged in re-embedding solution comprised of 4% (vol/vol) of 19:1 acrylamide/bis-acrylamide ,100 mM NaCl, 60 mM Tris ·HCl pH 8 and 0.2% (vol/vol) of TEMED for 20 min. The remaining re-embedding solution was kept on ice and further supplemented with 0.2% (wt/vol) ammonium persulfate just before casting the nonexpandable polyacrylamide gel. The expanded polyacrylate gels were transferred to silanized coverslips coated with fluorescent light yellow fiducial beads (Spherotech FP-0245-2), dried with KimWipes and added 100 µL re-embedding gel solution with ammonium persulfate. The samples were then put in a nitrogen chamber, covered with a GelSlick treated glass plate to facilitate

polyacrylamide gel polymerization at room temperature for 1-2 hr. The coverslip and the glass plate were again separated by using a razor. The samples were either used for MERFISH measurement immediately or stored in 2 × SSC containing 0.1% (vol/vol) murine RNase inhibitor at 4°C for no longer than a week. Gel embedding and tissue clearing for unexpanded human and mouse brain sections were performed as described previously (*17*).

<u>MERFISH imaging</u>

Human brain MERFISH imaging were carried out on the imaging platform as described previously (*15*). In the first round we imaged the cell nucleus stained with DAPI and poly-dT labeled readout probe complementary to the poly-dT LNA anchor probe. We then performed 16 rounds of three-color MERFISH imaging for the both 4,000- and 250-gene measurements of human tissues. Both DAPI and poly-dT were imaged at six focal planes separated by 1 μm in z. Each MERFISH round consisted of readout probe hybridization (10 min), washing (5 min), and imaging of ~1,000 tiled fields of view (FOV) (220 μm x 220 μm per FOV), readout fluorophore cleavage by TCEP (15 min), and rinsing with 2 x SSC (5 min). For each round, images were acquired with 750-nm, 650-nm, and 560-nm illumination at six focal planes separated by 1 μm in z to image the readout probes in addition to a single z-plane image with 405-nm illumination to image the fiducial beads on the glass surface for image registration. Specifically, the readout probes staining was done by flowing 10 nM (each) readout probes in hybridization buffer composed of 2 × SSC, 10% (vol/vol) ethylene carbonate (Sigma-Aldrich, E26258), 0.1% (vol/vol) murine RNase inhibitor (NEB), 0.5% (vol/vol) Triton X-100 and 0.4% (vol/vol) TWEEN 20 in nuclease-free water, following by washing with a wash buffer containing 2× SSC and 10% (vol/vol) ethylene carbonate in nuclease-free water. TWEEN 20 can passivate coverslip surface and reduce non-specific readout probe binding. Readout probe imaging was then performed in imaging buffer containing 5 mM 3,4- dihydroxybenzoic acid (Sigma), 2 mM trolox (Sigma), 50 μM trolox quinone, 1:500 recombinant protocatechuate 3,4-dioxygenase (rPCO; OYC Americas), 1:500 Murine RNase inhibitor, and 5 mM NaOH (to adjust pH to 7.0) in 2×SSC. After imaging, dyes on the readout probe were removed by flowing in cleavage buffer comprising 2 × SSC and

50 mM of Tris (2- carboxyethyl) phosphine (TCEP; Sigma) to cleave the disulfide bond connecting dyes to the probes. The imaging buffer and hybridization buffer were changed every 12-24 hours to prevent the decay of imaging quality. MERFISH imaging of mouse brain sections were performed as described previously (*17*).

<u>MERFISH decoding</u>

To align the 3-dimensional x, y, z image stacks for all 48-bit MERFISH measurements, we calculated the offset between the corresponding high-pass filtered fiducial bead images with subpixel resolution by finding the peak of the image cross-correlation and applied these offsets to align the image stacks. To identify the RNA transcripts in the registered image stacks, we modified our previously published pixel-based decoding algorithm (*15*, *45*). Our decoding framework included two stages: crude decoding and fine decoding. Each field of view (FOV) contained six z-planes and decoding between z-planes were independent. In the crude decoding, we first randomly selected one z-plane from each of the 100 randomly selected FOVs for pixel-based decoding. In short, we assigned barcodes to each pixel independently, then aggregated adjacent pixels with the same barcodes into putative RNA molecules and filtered the list of putative RNA molecules to enrich for correctly identified transcripts (*15*). In detail, to assign each pixel to one of the 4,140 (4,000 coding and 140 blank) barcodes, we compared the 48-dimensional intensity vectors measured for each pixel to the vectors corresponding to the 48-bit valid barcodes. To aid comparison, we first normalized intensity of each image in a bit by median intensity across the 100 random FOVs (using the randomly selected z-plane for each FOV) in the bit to eliminate the intensity variation between hybridizations and color channels. After intensity normalization, we further normalized intensity variations across pixels by dividing the 48-dimensional intensity vector for each pixel by its L2 norm, such that the magnitude in the 48-dimensional space was equal to 1 for each pixel. We similarly normalized each of the 4,140 valid 48-bit barcodes, such that the magnitude in the 48-dimensional space was equal to 1 for each valid barcode. To assign a barcode to each pixel, we identified the normalized barcode vector that was closest to the pixel's normalized intensity vector in the 48-dimensional space. We excluded pixels with distance larger than 0.65 away from any valid barcode in the first

step. To further reduce the chance of transcript misidentification, for each remaining pixel, we calculated two properties: the mean intensity, referred to as “pixel intensity”, and the distance between the normalized intensity vector of the corresponding pixel to the closest valid barcode, referred to as “pixel distance” hereafter. We anticipated that incorrectly identified barcodes would likely have a dimmer mean pixel intensity and a larger pixel distance as compared to correctly identified barcodes. To distinguish the valid pixel from the misassigned ones, we next trained a support vector machine (SVM) using pixels assigned to blank barcodes as negative control and pixels assigned to RNA-coding barcodes as positive ones. Because the RNA-coding pixels usually far outnumber the blank pixels, we down-sampled the RNA-coding pixels to the same number as the blank pixels to avoid sample imbalance. Using the trained SVM, we next predicted the probability of a pixel being a valid RNA-coding (positive) barcode. As expected, the pixels with dimmer pixel intensity or large pixel distance were predicted with lower probability while bright pixels with small pixel distance were assigned with a larger probability.

In the stage of fine decoding, we used the SVM classifier trained, and scaling factors estimated during the crude decoding to decode the rest of the z-planes. We first filtered any pixels that have a pixel distance greater than the distance cutoff (0.65). For the remaining pixels in each z-plane, we then assigned barcode ID to each pixel and grouped adjacent pixels if they were assigned to the same barcode ID to create a list of putative RNA molecules. We measured the confidence of the identified molecules by calculating the probability of at least one pixel being valid as following:

$$P(barcode_i) = 1 - \prod_{k=1}^{K} (1 - Pixel_k)$$

in which K is the total number of grouped adjacent pixels assigned to $barcode_i$ for this molecule and $Pixel_k$ represents the probability of pixel k is assigned to i-th barcode $barcode_i$ determined by SVM classifier. To avoid overfloating, instead of calculating the probability, we computed the log likelihood of the barcode:

$$L(barcode_i) = \sum_{k=1}^{K} \log(Pixel_k)$$

We next ranked all identified barcodes based on the barcode likelihood $L(barcode_i)$ and determined the likelihood cutoff that resulted in a gross barcode misidentification rate of 5%, where the gross barcode misidentification rate was estimated as (the mean count per blank control barcode) / (the mean count per RNA-coding barcode). Decoding of the MERFISH images of mouse brain samples were performed as described previously (*17*).

MERFISH image segmentation

We performed cell segmentation using co-staining of DAPI and polyA RNA as demonstrated previously for MERFISH analysis (*44*). Using a deep learning-based cell segmentation algorithm (Cellpose) (*47*), we first segmented cell nuclei with the DAPI image with diameter parameter of 150 pixels in the "nuclei" mode. We segmented individual z-planes for each FOV. We then identified the centroid position of each segmented nucleus in each z-plane and connected the centroids across different z-planes if they were within distance of 2 μm in xy direction, representing the same nucleus imaged in different z-sections. Because each of these image operations was applied to the full z-stack of images for each FOV, the generated nuclei segmentation boundaries were naturally in 3D. This algorithm was able to largely address the situation in which two adjacent cells partially overlapped along the xy direction.

We next segmented cell soma by polyA RNA image also using Cellpose with diameter parameter of 200 in a "cytoplasm" mode. We then used the segmented nuclei as seeds and segmented cell bodies as a mask on image manifolds established by polyA RNA image and performed watershed algorithm. In this manner, we assigned unique IDs for each cell body based on its unique the nuclear seed. Nuclei and cell bodies were assigned consistent cell IDs for downstream analysis. We performed watershed for individual z planes for each FOV to create the 3D segmentations.

Cloud-based massively parallel MERFISH decoding

Because MERFISH decoding is independent between z-planes, therefore, the decoding procedure can be divided into thousands of small independent tasks. This allowed us to speed up decoding of large-scale MERFISH datasets by leveraging the power of cloud computing platform. We set up an auto-scale SLURM cluster on Google cloud platform by following the instruction (https://cloud.google.com/architecture/deploying-slurm-cluster-compute-engine). The SLURM cluster had access to two types of compute engines including n1-standard-4 (4 vCPUs, 15 GB memory) and n1-standard-16 (16 vCPUs, 64 GB memory), up to 1,000 vCPUs and 2TB Standard persistent disk. n1-standard-4 machines with limited memory was used for decoding thousands of z-planes in parallel. n1-standard-16 was used for more memory-consuming tasks such as creating global mosaic image and barcode filtering. In both cases, preemptible nodes were used to reduce the cost. The decoding pipeline was managed using Snakemake - a tool to create reproducible and scalable data analyses (*48*). MERFISH data was stored in the Google bucket (STANDARDED) with unlimited storage that allowed the SLURM cluster to access.

Unsupervised clustering of 4,000-gene human MERFISH data

Because the cell type marker genes were identified using single nucleus SMART-seq data (*8*), in this study, we used the RNA molecules identified in the nucleus for clustering analysis. With the nucleus-by-gene matrix, we first preprocessed the matrix by several steps. The segmentation approach described above generated a small fraction of putative "nuclei" with very small total volumes or low RNA abundance due to segmentation artifacts, as well as some cells that overlapped in 3D and were not properly separated. We first filtered any cells with segmentation appeared in less than three z-planes to avoid spurious segmentations. Second, we filtered cells whose centroid positions were within 100 pixels to the edge of a FOV to avoid edge effect. Third, we removed the segmented "cells" with a total area across all z-planes that was either less than 300 μm^2 or larger than 6000 μm^2. Fourth, we calculated the RNA molecule density in each nucleus and filtered out instances with density less than 0.1

molecule / μm^2 which was closed to the molecule density outside the cell body, likely representing “empty nucleus” introduced by spurious segmentations or out-of-focus signal.

Using the remaining nuclei, we next performed clustering analysis. In detail, we first normalized the nucleus-by-gene count matrix using “scTransform”, a modeling framework for the normalization and variance stabilization of molecular count data from scRNA-seq experiments (*49*). To remove the differences in RNA counts due to the incompleteness of nucleus, we further normalized the RNA counts per cell by regressing out the imaged volume of each cell using “scTransform” by setting the “vars.to.regress” to the volume size of the nucleus. Next, we performed dimensionality reduction using principal component analysis (PCA), restricted to the 30 principal components (PCs) with the highest eigenvalues, and finally visualized using a 2D UMAP embedding (*50*). In the UMAP space, the batch effect between MERFISH experiments was further eliminated using Canonical Correlation Analysis (CCA) which was implemented in Seurat V3 (*51*). To identify transcriptionally distinct cell clusters, we performed graph-based Leiden (*52*) community detection (k=15; resolution=0.5) in the 30 PCs-space initially.

We first annotated identified clusters to major cell types based on the expression of canonical marker genes (**fig. S5**). Next, we performed separate clustering of inhibitory neurons and excitatory neurons from MERFISH data and SMART-seq data independently using the Single-Cell Consensus Clustering method (SC3) (*53*) to examine the correspondence between the two data modalities (**fig. S6**). We next determined the excitatory and inhibitory neuronal clusters by integrated analysis of the SMART-seq data (*8*) and MERFISH data (**fig. S7, A and B**). In detail, we performed normalization using “scTransform” for MERFISH and SMART-seq independently. ScTransform automatically selected 2,000 variable genes in both datasets as “anchors” for integration. Second, we used the selected anchors and CCA to generate the joint low-dimensional embedding space between MERFISH and SMART-seq data. In the joint space, we identified clusters using graph-based clustering method (Leiden). A key

parameter in Leiden is resolution, a higher resolution usually resulting in more clusters. How to choose the optimal resolution is described with more details below. First, for a clustering result generated by a given resolution, we trained a KNN classifier in the joint embedding space on 80% of the dataset and then estimated the prediction accuracy on the remaining 20%. We conducted this five times (also known as five-fold cross validation) to estimate the averaged prediction accuracy. As expected, we observed a decrease in prediction accuracy with increasing resolution, suggesting "over splitting" of clusters. In this study, we chose the resolution that yield 3% misidentification rate. To avoid the errors introduced by the integration algorithm, we next filtered any "outlier" clusters that were mostly identified by one dataset. In detail, for each cluster, we calculated the ratio between cell proportions determined by SMART-seq and MERFISH and then normalized these ratios to z-score across all clusters. We removed any clusters whose z-score was larger than 3 or smaller than -3. Finally, for each cluster, we performed differential expression (DE) analysis using Wilcoxon Rank Sum test in MERFISH and SMART-seq data separately (P-value < 1e-2; fold-change > 1.2). A cluster which failed to find a DE gene in both MERFISH and SMART-seq was merged with the closest clusters in the joint embedding space. This was repeated until no clusters could be merged anymore. 4000-gene MERFISH data from all MTG and STG sections were used for integrated MERFISH and SMART-seq analysis of neuronal clusters.

For clustering of non-neuronal cells, we used 4,000-gene MERFISH data alone (**fig. S7C**), because these cells were depleted in SMART-seq dataset. We first performed unsupervised clustering of the non-neuronal cells from neurosurgical MTG sections only, because the detected RNA copy numbers per cell were lower in the postmortem STG sections. We then performed supervised classification of non-neuronal cells in the postmortem STG data based on annotations from in the neurosurgical MTG data, using an approach similar to that described below for supervised classification of 250-gene MERFISH data.

<u>Supervised classification of 250-gene human MERFISH data</u>

To make use of our 250-gene MERFISH datasets, we performed supervised classification to predict the cluster labels for cells measured in the 250-gene experiments at the cluster level for neurons and subclass level for non-neuronal cells based on the annotation of the 4,000-gene MERFISH datasets. We performed normalization using “scTransform” for 4,000-gene and 250-gene MERFISH dataset separately. In this case, ScTransform selected 2,000 variable genes for 4,000-gene MERFISH dataset and 250 variable genes for 250-gene MERFISH datasets. Second, we used CCA to generate the joint low-dimensional embedding space between 4,000- and 250-gene datasets. We next applied the neighborhood-based classifier to make a classification of labels for cells in 250-gene dataset. Briefly, we assigned a cluster label to each cell in 250-gene dataset based on the cluster labels of its neighboring cells in the annotated 4,000-gene dataset. The contribution of the neighboring cells in the 4,000-gene dataset was weighted by their distances to the query cell in the 250-gene dataset. In detail, for each cell $\boldsymbol{c}$ in 250-gene dataset, we identified the nearest $\boldsymbol{s}$ ($\boldsymbol{s}$=50) cells in the 4,000-gene dataset in the joint embedding space in the first 30 dimensions. For each cell a_i among the $\boldsymbol{s}$ cells, we calculated the Euclidean distance $\mathrm{dist}(c, a_i)$ between the query cell $\boldsymbol{c}$ and reference cell a_i. This distance was then weighted based on the distance to the s-th reference cell in the 4,000-gene dataset:

$$D_{c,i} = 1 - \frac{\mathrm{dist}(c,a_i)}{\mathrm{dist}(c,a_s)}$$

The weighted distance was converted to weighted similarity:

$$S_{c,i} = 1 - e^{\frac{-D_{c,i}}{(\frac{2}{sd})^2}}$$

where *sd* is set to 1 by default. Finally, we normalized the similarity across all $\boldsymbol{s}$ anchors:

$$W_{c,i} = \frac{S_{c,i}}{\sum_1^{j=s} S_{c,j}}$$

Hence $W_{c,i}$ is the weighted similarity between query cell $\boldsymbol{c}$ and reference cell a_i.

Using the weighted similarity, we next predicted the cluster label for query cell $\boldsymbol{c}$. In detail, let $\mathbf{L} \in \boldsymbol{\mathcal{R}}^{k \times t}$ be the binary label matrix for k cells with t clusters. $L_{i,j} = 1$ indicates that the class label for i-th 4,000-gene cell within the 50 nearest neighbor is j-th cluster. The row sum of $\mathbf{L}$ must be 1, because each 4,000-gene cell can only be assigned to one cluster label. We then compute label predictions for query cells as $\mathbf{P}^l$:

$$\mathbf{P}^l = \mathbf{WL}$$

The resulting $\mathbf{P}^l$ is a probability matrix, $P^l_{i,j}$ indicates the probability of a cell i assigned to cluster j. Each cell in the 250-gene measurement was assigned to the cluster label that had the maximum probability. Cells with the maximum assignment probability less than 0.4 was removed from downstream analysis.

Unsupervised clustering of mouse MERFISH data

Cell classification for the mouse VIS, AUD and TEa regions were performed by integrated analysis of the 242-gene mouse MERFISH data and SMART-seq data, in the same manner as we did for 4,000-gene human MERFISH data.

Cell-cell contact or proximity enrichment analysis

We defined two cells to be in contact or proximity if they were within 15 μm of each other, a distance that was close to the size of the somata of cells in both human and mouse cortex (**fig. S12**). We calculated the contact or proximity frequency between two cell types (i.e. A and B) by counting the number of cell pairs between A and B whose distance were below this 15 μm threshold in the 2D spatial map. We noticed and corrected the following biases in the interaction map. First, the cells in the regions of high cell density usually resulted in higher contact or proximity frequency as compared to those in the sparse regions. Second, cell types of similar laminar organization resulted in higher contact or proximity frequency. To correct for these biases, we randomized the cell position within a short-range distance to disrupt the proximity

between neighboring cells while largely preserves the higher-order laminar organization and local cell density (**fig. S13**). In detail, we randomized the position of each cell within a radius of 100 μm of its measured position and determined the contact or proximity frequency between any two cell types, and we performed such spatial permutation 1,000 times to generate a null distribution. We then determined the fold change between the measured contact or proximity frequency and the mean expected frequency from the 1000 spatial permutations. By calculating the z-score for the observed contact or proximity frequencies based on the null distribution, we obtained a P-value for the significance of contact or proximity enrichment between two cell types. Finally, P-values were corrected using the Benjamini-Hochberg (BH) procedure. To examine the effect of permutation radius, we performed the spatial permutation test with different radii from 35 μm to 150 μm and observed similar results (**fig. S14**). To examine the effect of cell density on cell-cell contact or proximity enrichment analysis, we down-sampled the mouse cortex data to lower cell densities, the lowest being similar to the cell density of the human cortex, and observed similar cell-cell contact or proximity enrichment at these different cell densities (**fig. S15, A**). We also generated simulated data with and without cell-cell interactions at different cell densities and did not observe any appreciable effect of cell density on the determination of cell-cell contact or proximity enrichment (**fig. S15, B and C**).

As an alternative approach to determine whether two cells were in contact, we used the segmented cell boundaries. Two cells with segmented boundaries algorithmically touching each other (within 300 nm) were considered in contact. We then calculated the contact frequency between two cell types and determined how much and how significant this frequency was greater than the expected frequency by comparing with spatial permutations that disrupt the proximity between neighboring cells (**fig. S13**), as described above.

<u>Ligand-receptor analysis</u>

To determine the enrichment of a ligand–receptor pair (L_i-R_i) in two cell types (i.e. A and B), we first identified cells pairs between A and B that were contacting or in

proximity as described in the section above. For each contacting / proximal cell pairs, we calculated the product of the normalized expression (logCPM) of the ligand (L_i) by one cell and receptor (R_i) by another cell, and determined the sum of this product across all contacting cell pairs as the ligand-receptor score for cell types A and B. We then determined the enrichment significance using empirical shuffling. In each shuffling, we randomly selected the same number of cell pairs from A and B that satisfy the following criterion: each cell in cell type A having no cell in cell type B that is within 15 µm, and calculated the ligand-receptor score for $L_i - R_i$ as described above. We repeated this for 1,000 times to obtain the null distribution. Using one-tailed Z-test, we obtained a P-value for the significance and adjusted the P-values using the BH procedure. The fold change was determined as the observed ligand-receptor score over the average of the expected ligand-receptor scores derived from the empirical shuffling.

Single-molecule FISH

The single-molecule FISH (smFISH) primary probes were designed as described above for MERFISH encoding probes and were purchased from Integrated DNA Technologies (**Table S6**). The human tissue slices were fixed by treating with 4% PFA in 1×PBS for 10-15 minutes and were washed three times with 1×PBS and stored in 70% ethanol at 4°C for overnight to permeabilize the tissues. After permeabilization, tissue slices were exposed under the multi-band light emitting diode arrays for three hours to eliminate the autofluorescence background (*13*). The samples were incubated for 30 min in wash buffer comprising 2× SSC (Ambion), 30% (vol/vol) formamide (Ambion), and 0.1% (vol/vol) murine RNase Inhibitor (New England Biolabs). The sample was then stained with a drop of 100 µL of smFISH primary probes (~10 nM per primary probe) and acrydite-modified poly(dT) locked nucleic acid (LNA) probes (2 µM) in encoding hybridization buffer. The samples were then incubated in a cell culture incubator at 37 °C for ~24 hr, followed by washing with encoding wash buffer twice at 47 °C for 30 min. All tissue samples were gel-embedded and cleared to remove fluorescence background as we previously described (*17*). Briefly, the samples were embedded in a thin polyacrylamide gel and were then treated with a digestion buffer of 2% v/v sodium dodecyl sulfate (SDS; AM9823, Thermo Fisher), 0.5% v/v Triton X-100 (X100, Sigma)

and 1% v/v proteinase K (P8107S, New England Biolabs) in 2× SSC for 36–48 hr at 37 °C. After digestion, the samples were washed in 2× SSC for 30 min for a total of four washes before imaging. The smFISH primarily probes were then detected using readout probes in sequential rounds of multi-color imaging to image the ligand, receptor and cell type marker genes (7 sequential rounds of three-color FISH imaging to measure 19 genes). Both smFISH probes and DAPI were imaged at six focal planes separated by 1 μm in z.

To identify the RNA molecules, we first performed high-pass-filter to the smFISH images to remove the cellular background. Filtered images from all six z-planes were maximum projected to a single image. Second, we used thunderSTORM (*54*) to identify the molecule localizations. To filter out the spurious localizations due to the off-target (non-specific) binding of probes, we removed the localizations with low intensity. In detail, we found that the logarithm-transformed localization intensity clearly obeyed a bimodal gaussian distribution, with the low intensity distribution representing the non-specific binding and high-intensity distribution representing the specific, on-target binding. Therefore, we used bimodal gaussian distribution to estimate the mean and variance for the low- and high-intensity peaks, and filtered out localizations belonging to the low-intensity peak. We also identified abnormally bright and large localizations that were likely due to lipofuscin in the human brain and filtered out these localizations.

We next determined the excitatory neurons and microglial cells based on the expression of corresponding marker genes – *SLC17A7* and *SELPLG.* The L2/3 and L4/5 IT excitatory neurons were further identified based on the cortical layer morphology as L4 has higher cell density and L1 has no excitatory neurons. Finally, the ligand-receptor enrichment was determined in the same manner as described in the previous section.

<u>Replicates and statistics details</u>

For human cortex analysis, we performed 4,000-gene MERFISH imaging on five sections from neurosurgical MTG samples (from two male individuals, 36 and 32 years old; donor ID: H18.06.006 and H22.26.401) and five sections from postmortem STG

samples (from two male individuals, 29 and 42 years old; donor ID: H19.30.001 and H20.30.001). All 4,000-gene MERFISH experiment were performed on expanded tissue sections. We also performed 250-gene MERFISH measurements on two expanded tissue sections from two donors (H18.06.006 and H19.30.001) to assess the detection efficiency of the 4,000-gene measurements from the same donors. We additionally performed 250-gene measurements on three unexpanded sections from the other two donors. For mouse cortex analysis, we imaged four sections from two mouse brains (male, 57-63 days old) without sample expansion.

This study did not require the allocation of samples into different experimental groups. Hence, randomization and blinding were not needed for this study. We did not exclude any data from consideration. All MERFISH images were included in the primary analysis.

Some of the human MTG and STG sections did not cover the entire cortical depth from L1 to white matter, we thus only considered sections with a relatively complete coverage of the cortical depth from L1 to white matter for cell-type composition analyses in Fig. 1, D and E, and cortical depth distribution analyses in Figs. 2B and 3C, and figs. S9 and S10, including both 250-gene and 4,000-gene measurements unless otherwise mentioned. Because the non-neuronal cells in 250-gene measurements were classified only at the subclass level, the cortical depth distribution analyses of non-neuronal clusters in Fig. 2B and fig. S9 were derived from the 4,000-gene datasets only. Even for these sections with a relatively complete coverage of the cortical depth, the superficial layer L1 and the white matter tended to be incomplete. For soma contact or proximity analyses, 250-gene and 4,000-gene data from all tissue sections were considered, regardless of whether the sections had relatively complete coverage of the cortical depth. For ligand-receptor analysis, only 4,000-gene data were considered.

Supplementary Figures

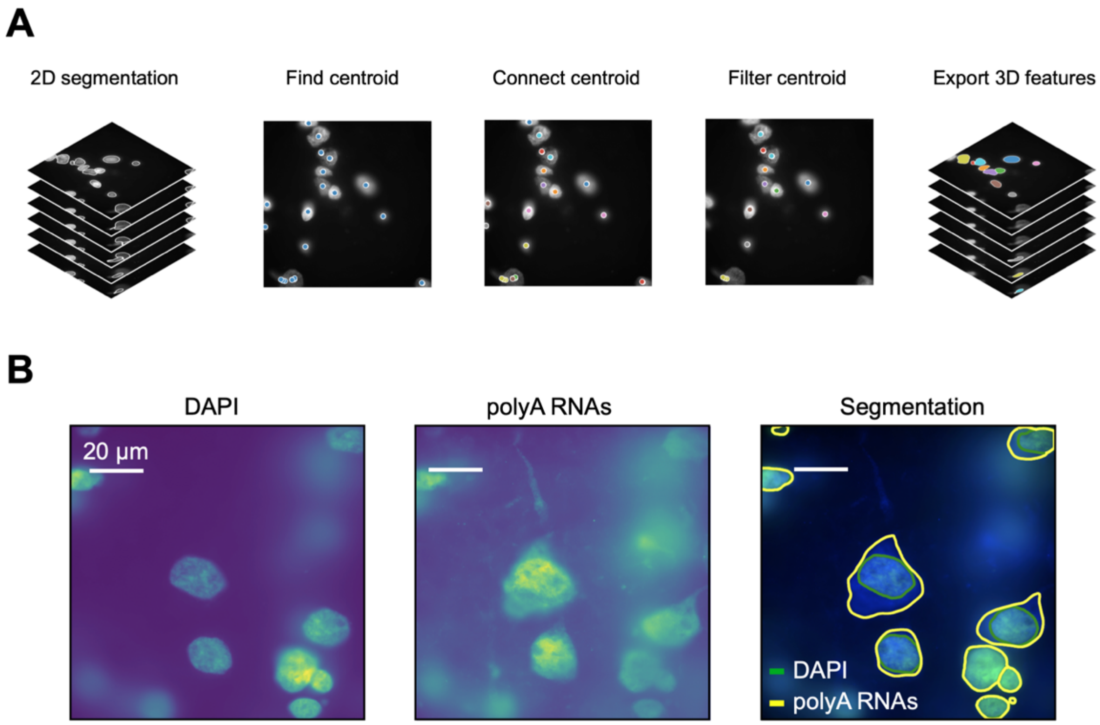

Fig. S1. Cell segmentation based on DAPI and polyA RNAs signals. (**A**) The schematic of 3D cell segmentation. Cell boundaries were first identified in each z-plane separately using deep learning-based segmentation algorithm (Cellpose) (*47*). The centroids of each 2D segmentations were connected between z-planes if their distance in xy was smaller than 2 μm. The connected centroids represent the same cell sampled in different z-planes. Cells that did not appear in at least three consecutive z-planes were likely due to segmentation errors and were filtered out. (**B**) Segmentation of cells with DAPI and polyA RNA co-stains in a single field-of-view. DAPI and polyA RNA images of the field-of-view are shown in the left and middle panels, respectively, and the right panel shows the segmentation boundaries for each cell and each nucleus marked in yellow and green, respectively.

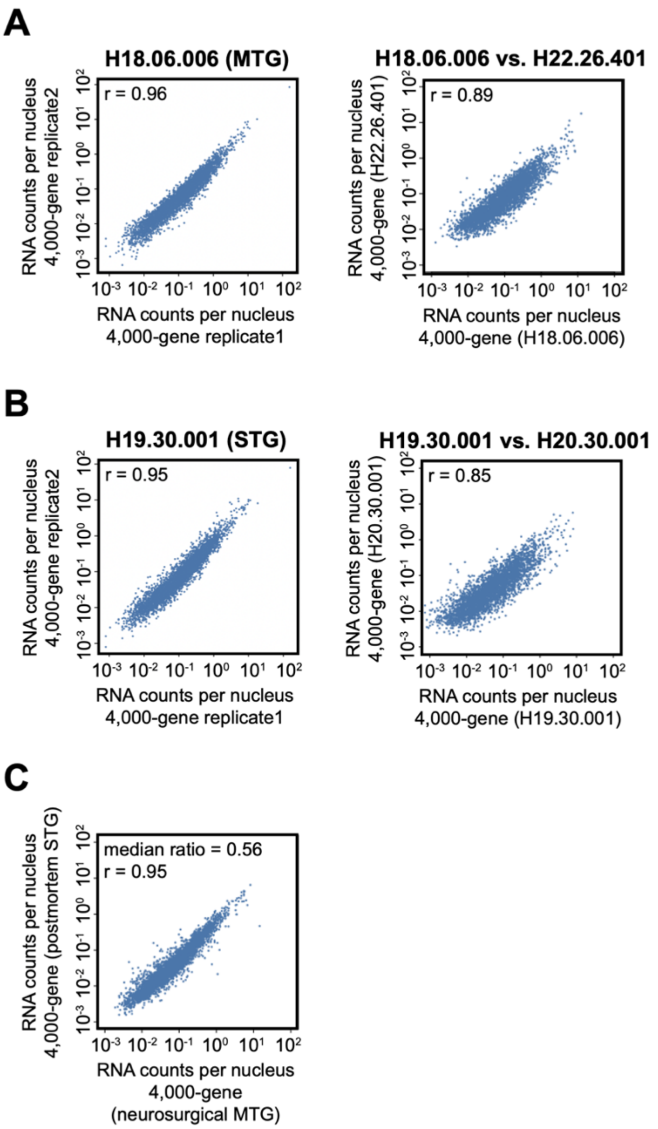

Fig. S2. Reproducibility of MERFISH data between replicates of human samples. (**A**) The scattered plots of the RNA copy number per nucleus for individual genes detected in the 4,000-gene MERFISH measurements between two example MTG slices of the same individual (left) and between MTG slices from two different individuals (right). H18.06.006: Male, 36 years old. H22.26.401: Male, 32 years old. (**B**) Same as (A) but for STG instead of MTG. H19.30.001: Male, 29 years old. H20.30.001: Male, 42 years old. (**C**) The average RNA copy number per nucleus for individual genes detected in the 4,000-gene MERFISH measurements of the neurosurgical MTG samples versus that of the postmortem STG samples. r stands for the Pearson correlation coefficient.

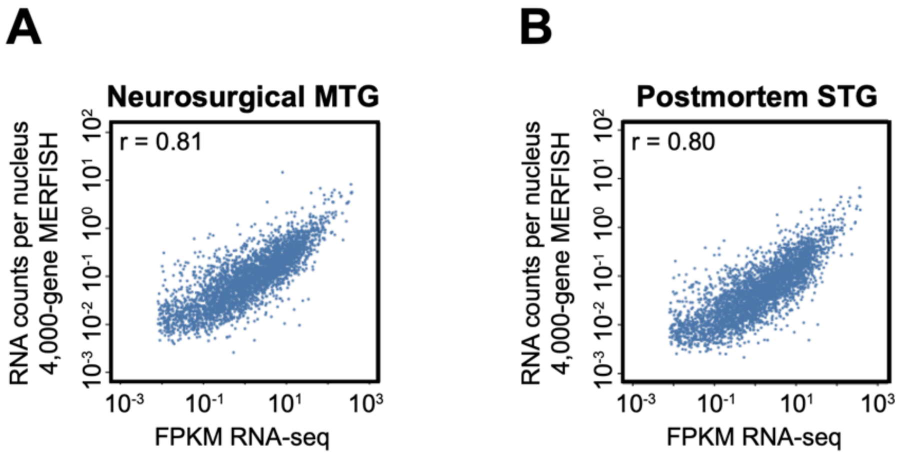

Fig. S3. Correlation between MERFISH data and bulk RNA sequencing data. (**A-B**) The RNA copy number per nucleus for individual genes detected in the 4,000-gene MERFISH measurements of the MTG (**A**) and STG (**B**) samples versus the FPKM from bulk RNA-seq. r stands for the Pearson correlation coefficient.

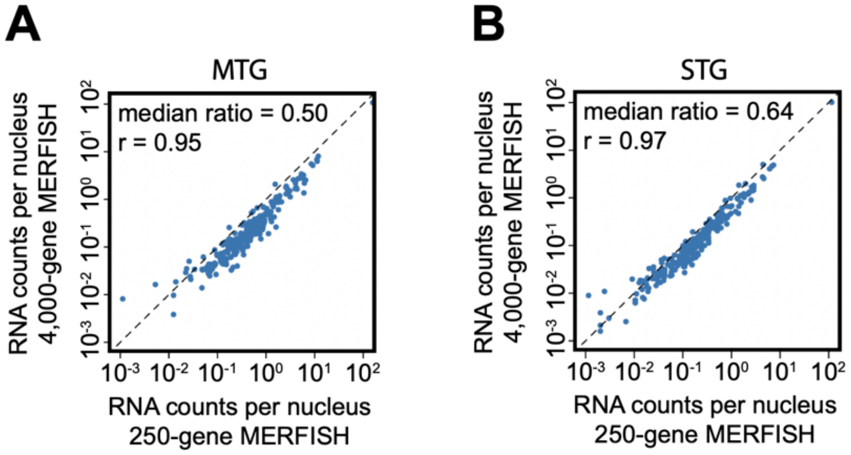

Fig. S4. Comparison of 4,000-gene and 250-gene MERFISH data. The average RNA copy number per nucleus for individual genes detected in 4,000-gene measurements are compared to that in the 250-gene measurements for the 250 genes that are shared in both measurements. (**A**) For the MTG samples (H18.06.006). (**B**) For the STG samples (H19.30.001). r stands for the Pearson correlation coefficient. Only donors measured with both 4,000-gene and 250-gene experiments on expanded sections are considered here.

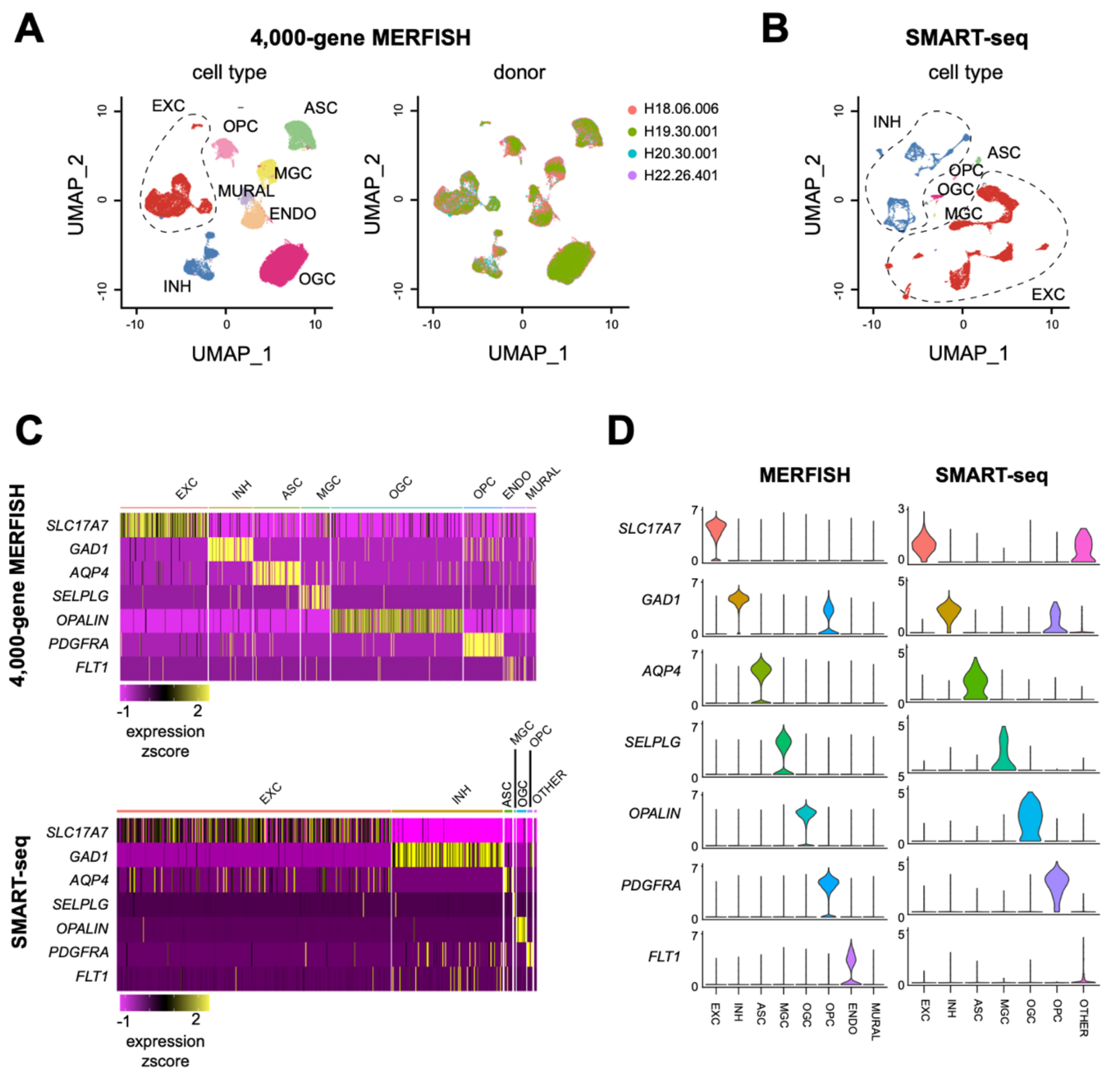

Fig. S5. Identification of inhibitory and excitatory neurons and non-neuronal cells by MERFISH and SMART-seq. (**A**) UMAP visualization of inhibitory and excitatory neurons and major subclasses of non-neuronal cells identified by 4,000-gene MERFISH data, colored by cell types (left) and donors (right). (**B**) UMAP visualization of cells from the previously reported SMART-seq data (*8*). (**C**) Heatmap and (**D**) violin plot showing the expression of marker genes for the major subclasses of cells described in (**A-B**) identified in MERFISH and SMART-seq data.

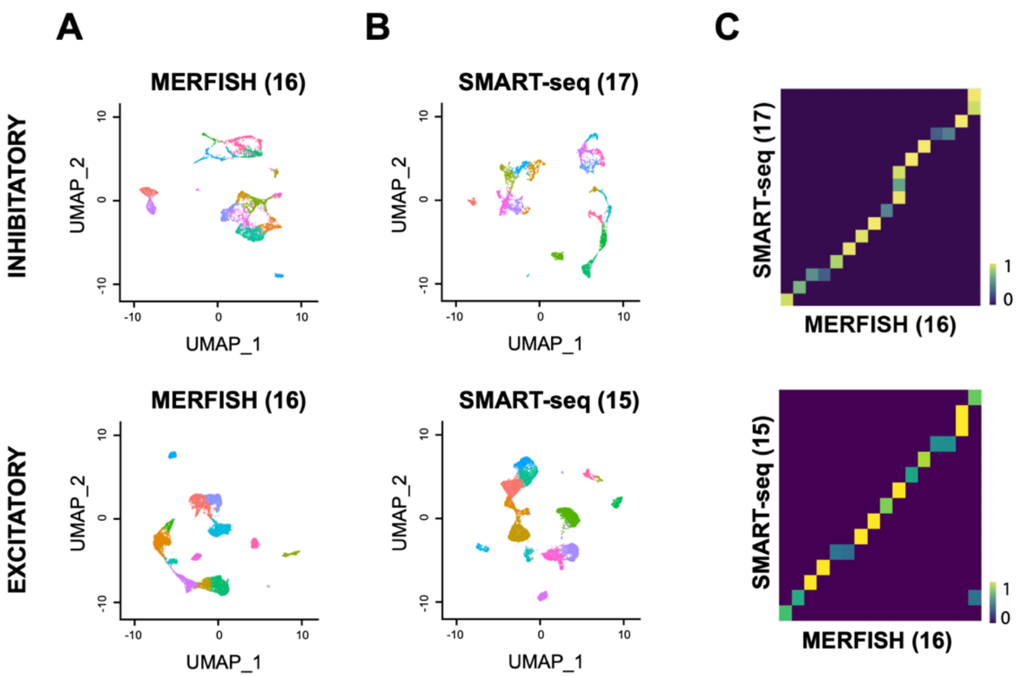

Fig. S6. Identification of inhibitory and excitatory neuronal clusters by MERFISH and SMART-seq separately. (**A**) UMAP visualization of inhibitory (top) and excitatory (bottom) neuronal clusters identified by 4,000-gene MERFISH. (**B**) UMAP visualization of inhibitory (top) and excitatory (bottom) neuronal clusters identified by SMART-seq. (**C**) Correspondence between the clusters determined by MERFISH and the clusters determined by SMART-seq for inhibitory (top) and excitatory (bottom) neuronal clusters. The clusters were determined by the Single-Cell Consensus Clustering method (*53*). To assess the correspondence between MERFISH and SMART-seq clusters, a k-nearest neighbor classifier was trained using the MERFISH dataset and used to predict a MERFISH cluster label for each cell in the SMART-seq dataset. Cells were grouped based on their SMART-seq cluster identity, and the fraction of cells from a given SMART-seq cluster that were predicted to have each MERFISH cluster label were plotted.

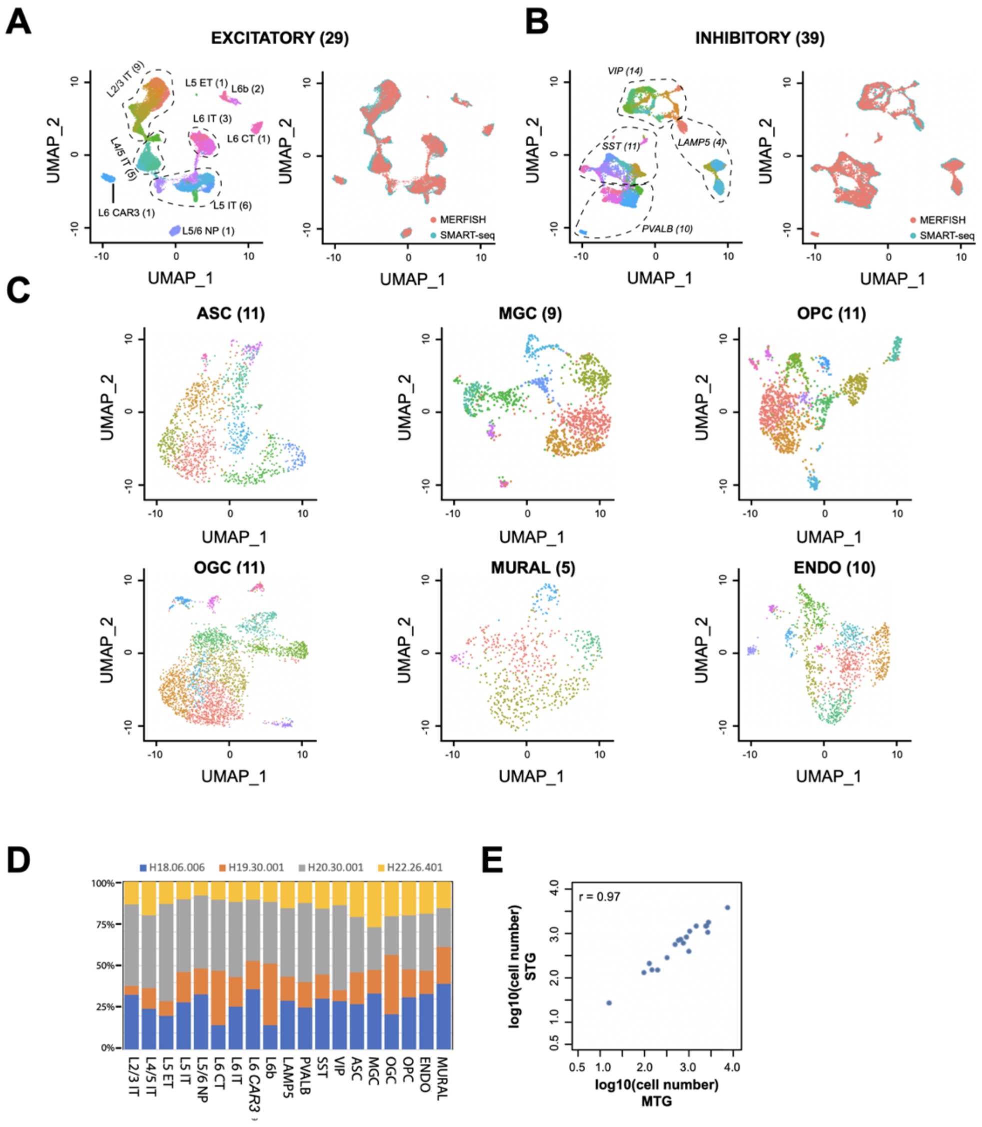

Fig. S7. Identification of inhibitory and excitatory neuronal clusters and non-neuronal clusters. (**A-B**) UMAP visualization of excitatory (**A**) and inhibitory (**B**) clusters identified by integrated analysis of the 4,000-gene MERFISH (all MTG and STG sections) and SMART-seq data. Cells were colored by the cluster labels (left) or different technological platforms (right). (**C**) UMAP visualization of non-neuronal clusters identified by 4,000-gene MERFISH data from neurosurgical MTG sections. (**D**) Cell-type contributions from the four donors. The percentage of cells in each subclass that are from individual donors are shown. We note that some of the samples did not cover the entire cortical depth from L1 to white matter, and hence exhibited variations in contributions across different subclasses. In addition, L5 ET, L5/6 NP, L6 CT and L6b

are low abundance cell types and hence could have higher variations due to lower statistics. (E) The cell number for each individual subclass identified in the MTG samples versus STG samples.

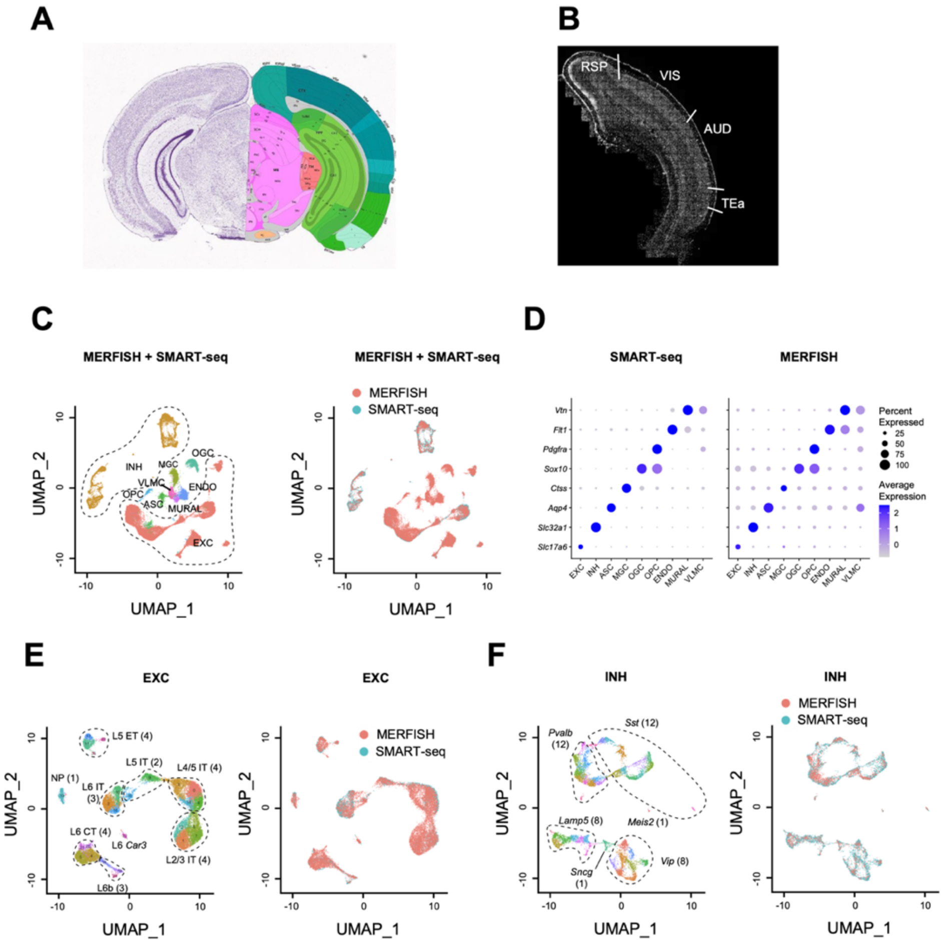

Fig. S8. MERFISH imaging and cell type identification of the mouse cortex. (**A**) Schematic of a mouse coronal section that contains the cortical regions for MERFISH imaging based on the Allen Common Coordinate Framework (CCFv2). (**B**) Mosaic DAPI image of the cortical region that contains the visual cortex (VIS), auditory cortex (AUD), temporal association area (TEa), and adjacent areas imaged by MERFISH. (**C**) The inhibitory and excitatory neurons and major subclasses of non-neuronal cells identified by integrated analysis of MERFISH and SMART-seq data. Cells were colored by subclasses (left) or technological platforms (right). (**D**) Dot plot displaying the expression of canonical marker gene in the major cell types shown in (**C**) for the SMART-seq (left) and MERFISH (right) data. The size and color shade of the dots corresponds to the percentage of cells expressing the indicated gene in each cluster and the average expression level, respectively. (**E-F**) The excitatory (**E**) and inhibitory (**F**) neuronal clusters identified by integrated analysis of the MERFISH and SMART-seq data. Cells were colored by cluster labels (left) or technological platforms (right).

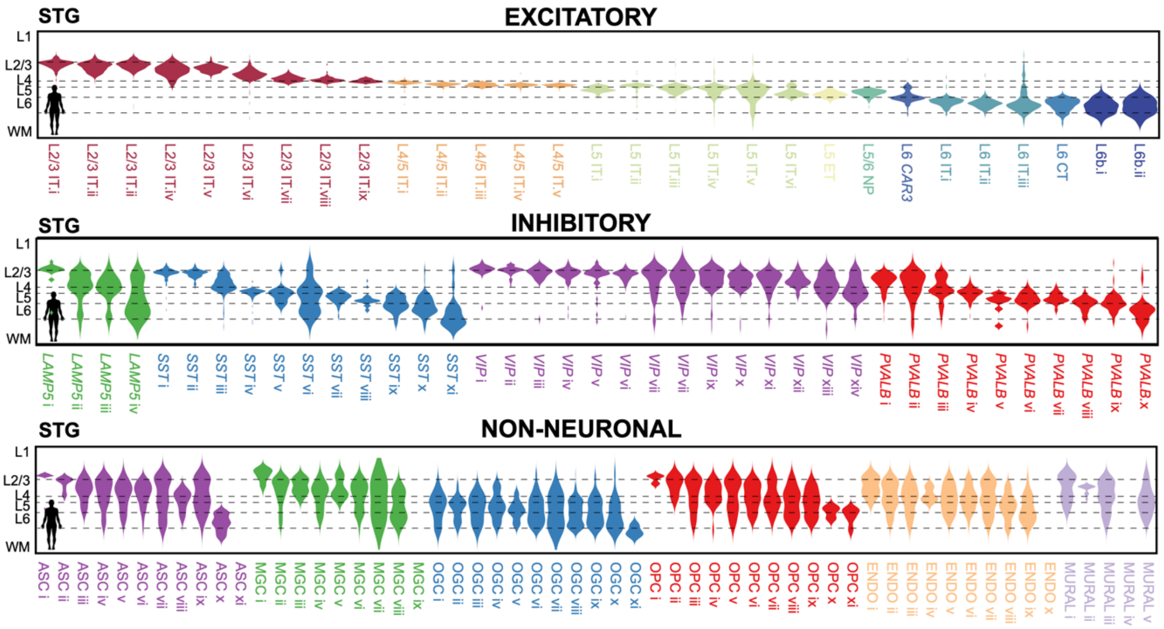

Fig. S9. Cortical-depth distributions of excitatory, inhibitory, and non-neuronal clusters in the human STG. Top: Excitatory neuronal clusters. Middle: Inhibitory neuronal clusters. Bottom: Non-neuronal clusters. The dashed grey lines mark the approximate layer boundaries. WM: white matter. The cortical-depth distributions of clusters with fewer than 10 cells (ASC xi, MGC ix, ENDO x, and MURAL iv) are not depicted. Only tissue sections that had a relatively completed coverage of the cortical depth from L1 to WM were included in the analysis here and in other cortical-depth distribution figures (Figs. 2B, 3C and fig. S10), as well as in the cell-composition figures (Fig. 1, D and E). Even in these sections, L1 and WM tended to be incomplete and hence the distributions in L1 and WM (in this and other cortical-depth distribution figures) could be affected by the incompleteness of these layers. For the non-neuronal cell distributions, only 4,000-gene MERFISH datasets were considered because the 250-gene data only classified cells at the subclass level.

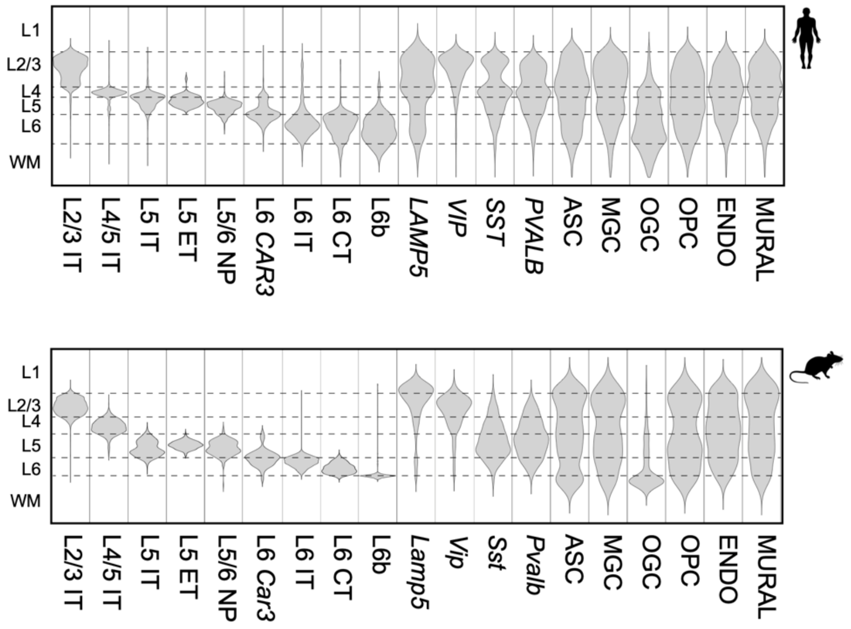

Fig. S10. Comparison of cortical-depth distributions of neuronal and non-neuronal subclasses between human and mouse. Top: Cortical-depth distributions of different subclasses of cells in the human MTG and STG. Bottom: Cortical-depth distributions of different subclasses of cells in the mouse cortex.

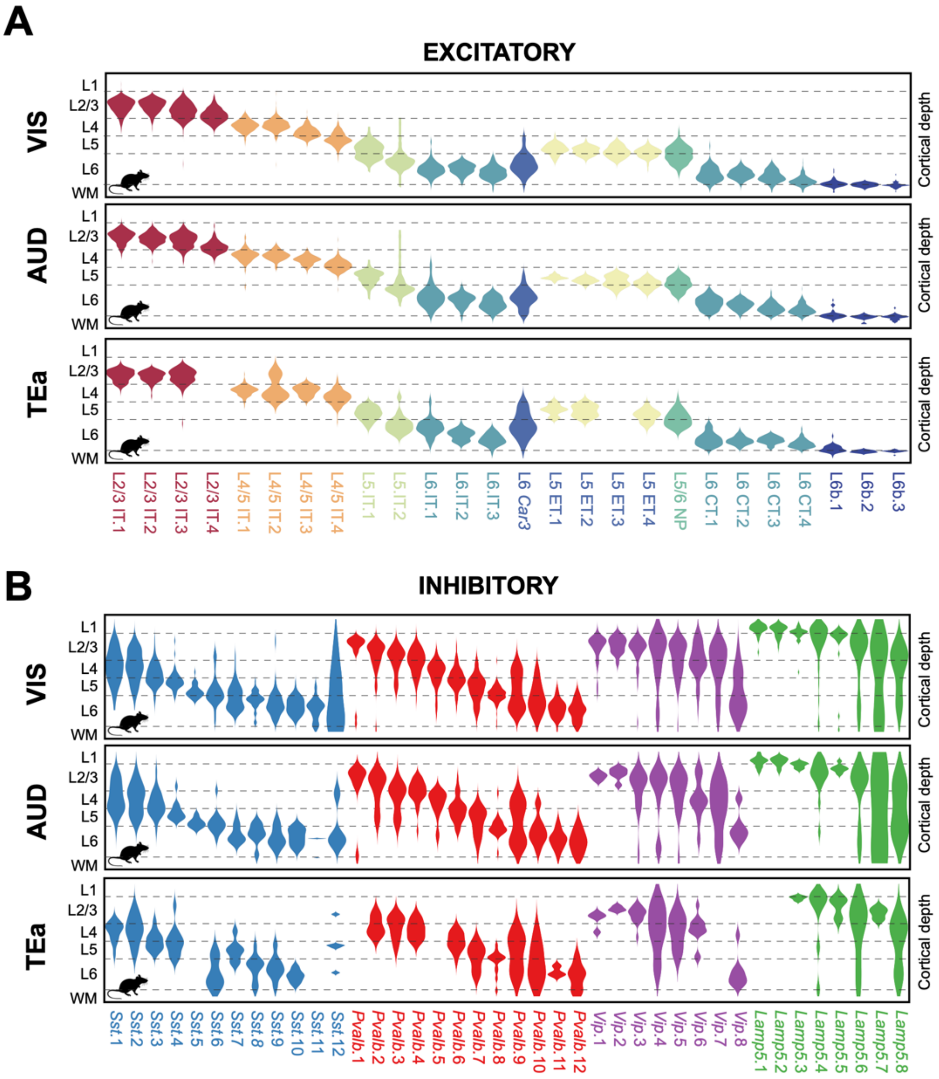

Fig. S11. Cortical-depth distributions of the neuronal clusters in different mouse cortical areas. (**A**) Excitatory neuronal clusters. (**B**) Inhibitory neuronal clusters. The cortical-depth distributions of clusters with fewer than 10 cells are not depicted.

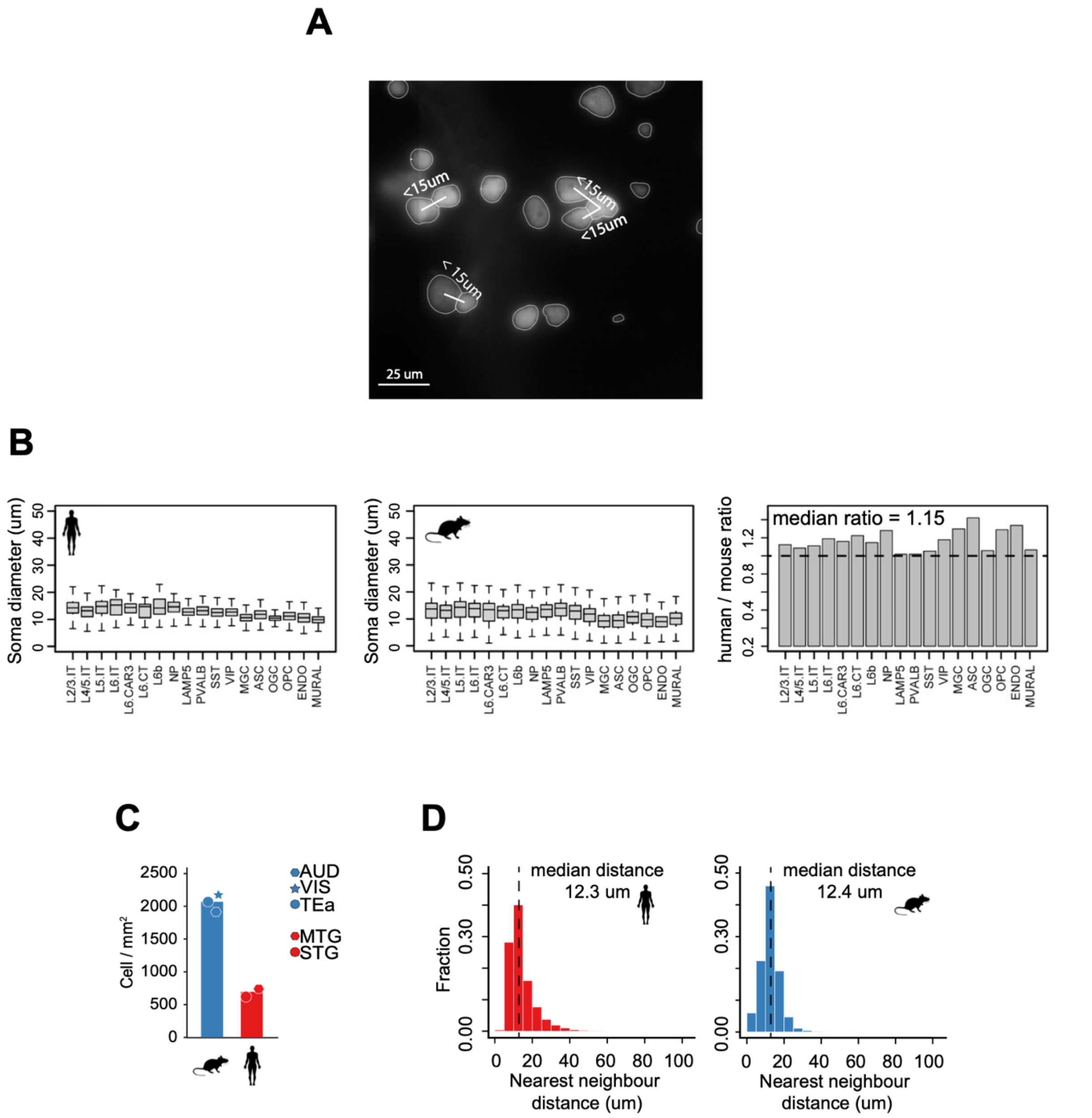

Fig. S12. Probing soma contact or proximity by centroid distance between cells. (**A**) Schematic for soma contact or proximity determination with a distance threshold between the centroid positions of neighboring cells. We used 15 μm as the distance threshold, which is close to soma sizes of both human and mouse samples, and we considered neighboring cells with distance smaller than 15 μm as being in contact or proximity. (**B**) Left and Middle: Soma sizes of different subclasses of cells in the human (left) and mouse (middle) cortex. Right: Human-to-mouse ratio of soma sizes in different subclasses. In the box plot, midline is the median, box edges are 75th and 25th

percentiles, and whiskers indicate 1.5 times the interquartile range. Soma diameter for each cell was determined by first identifying the z plane in which a cell has the largest area size and then calculating $2 \times \sqrt{area / \pi}$ as a proxy to the soma diameter. (**C**) Mean density of cells in the multiple human and mouse cortical regions examined. (**D**) Distributions of the centroid distances between nearest-neighbor cells in the human (left) and mouse (right) cortex.

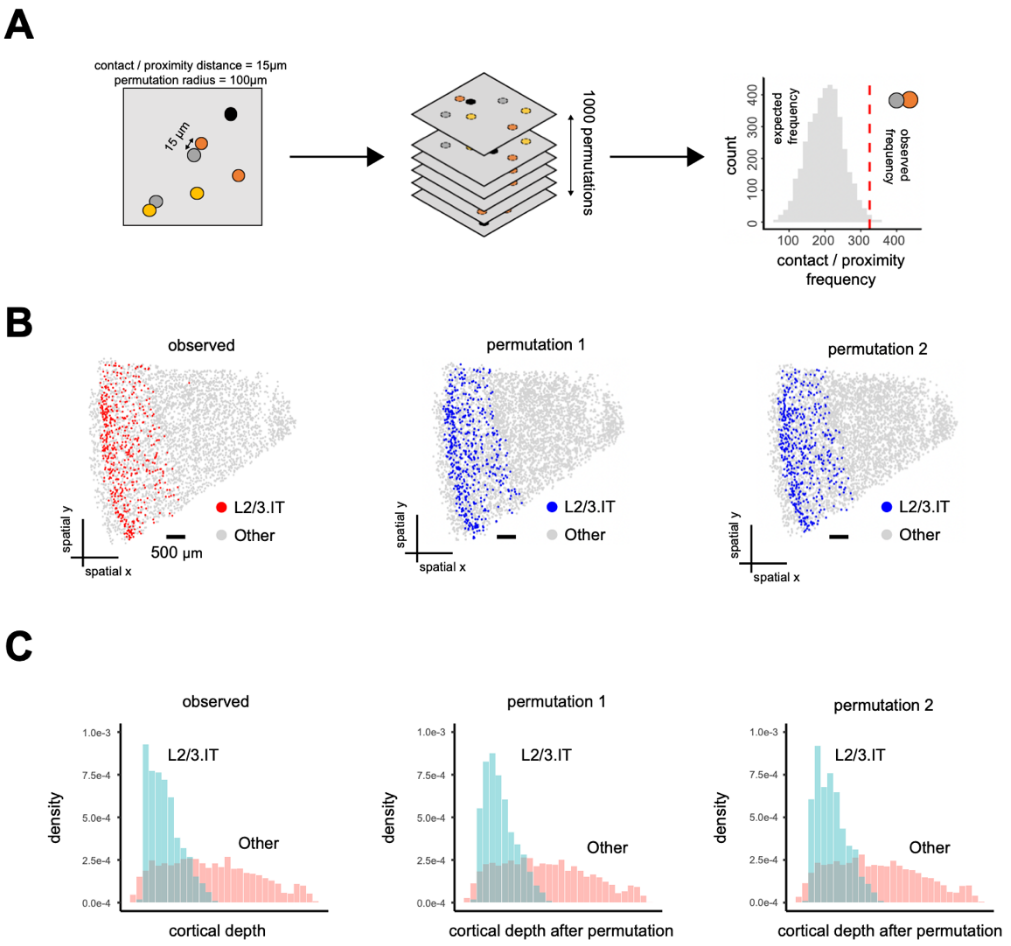

Fig. S13. Spatial permutations that disrupt the relationship between neighboring cells while preserving the laminar distribution and local density of each cell type. (**A**) Schematic of spatial permutation test that determines the significance of soma contact or proximity enrichment between cell types. Two cells were considered in contact or proximity if their nucleus centroids were within 15 μm in the imaging plane. Contact or proximity frequency between any two cell types was determined as the observed frequency. Then, spatial localization of each cell was randomized within a radius of 100 μm, unless otherwise mentioned. Expected contact or proximity frequency between any two cell types was determined in each permutation and such permutation was performed 1,000 times to obtain the distribution of expected contact or proximity frequencies. The significance of the enrichment of observed contact or proximity frequency over expected frequency was calculated by comparing the observed

frequency with the distribution of the expected frequencies using one-tailed Z-test and P-values were corrected to FDR (false discovery rate) using Benjamini-Hochberg Procedure. (**B**) Spatial map of L2/3 IT cells in a human MTG slice. (Left) Measured spatial map. (Middle and right) Two example spatial maps after the spatial permutations described in (**A**). (**C**) Cortical depth distributions of L2/3 IT cells (light blue) and other cells (light red) in the human MTG slice shown in (**B**). (Left) Measured cortical depth distributions. (Middle and right) Cortical depth distributions after the two example spatial permutations as described in (**A**).

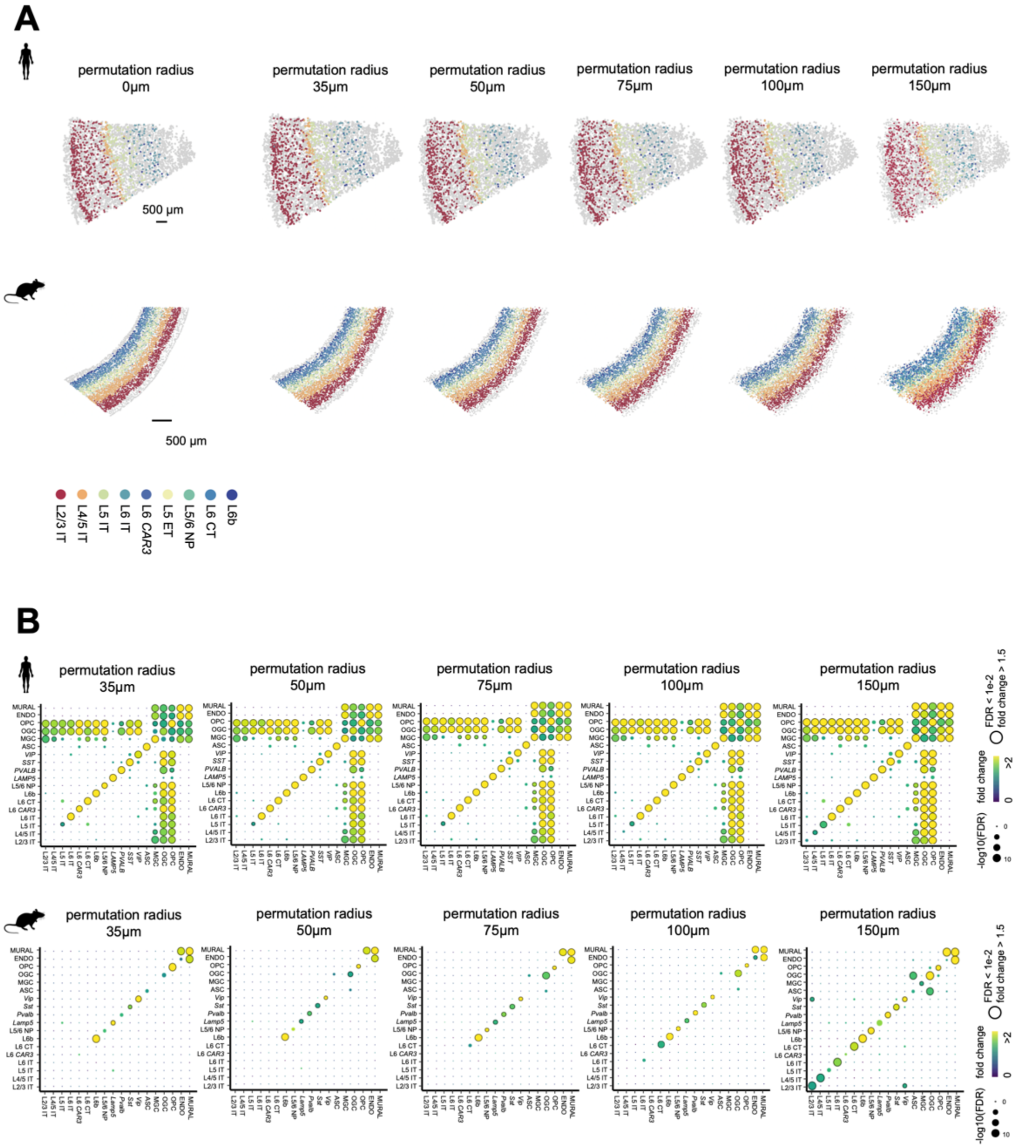

Fig. S14. The effect of permutation radius on soma contact or proximity enrichment analysis. (**A**) Spatial map of a human MTG slice (top) and a VIS-containing region in a mouse cortex slice (bottom) (permutation radius = 0) followed by examples of spatial maps after spatial permutations with different permutation radius ranging from 35 µm to 150 µm. Excitatory neurons were colored by their subclass labels and other cells were shown in grey. (**B**) Pairwise soma contact or proximity enrichment map for subclasses of cells in human (up) and mouse (bottom) cortex shown in dot plots for different permutation radius from 35 µm to 150 µm. The similarity between enrichment maps obtained with different permutation radii indicates the robustness of the permutation results.

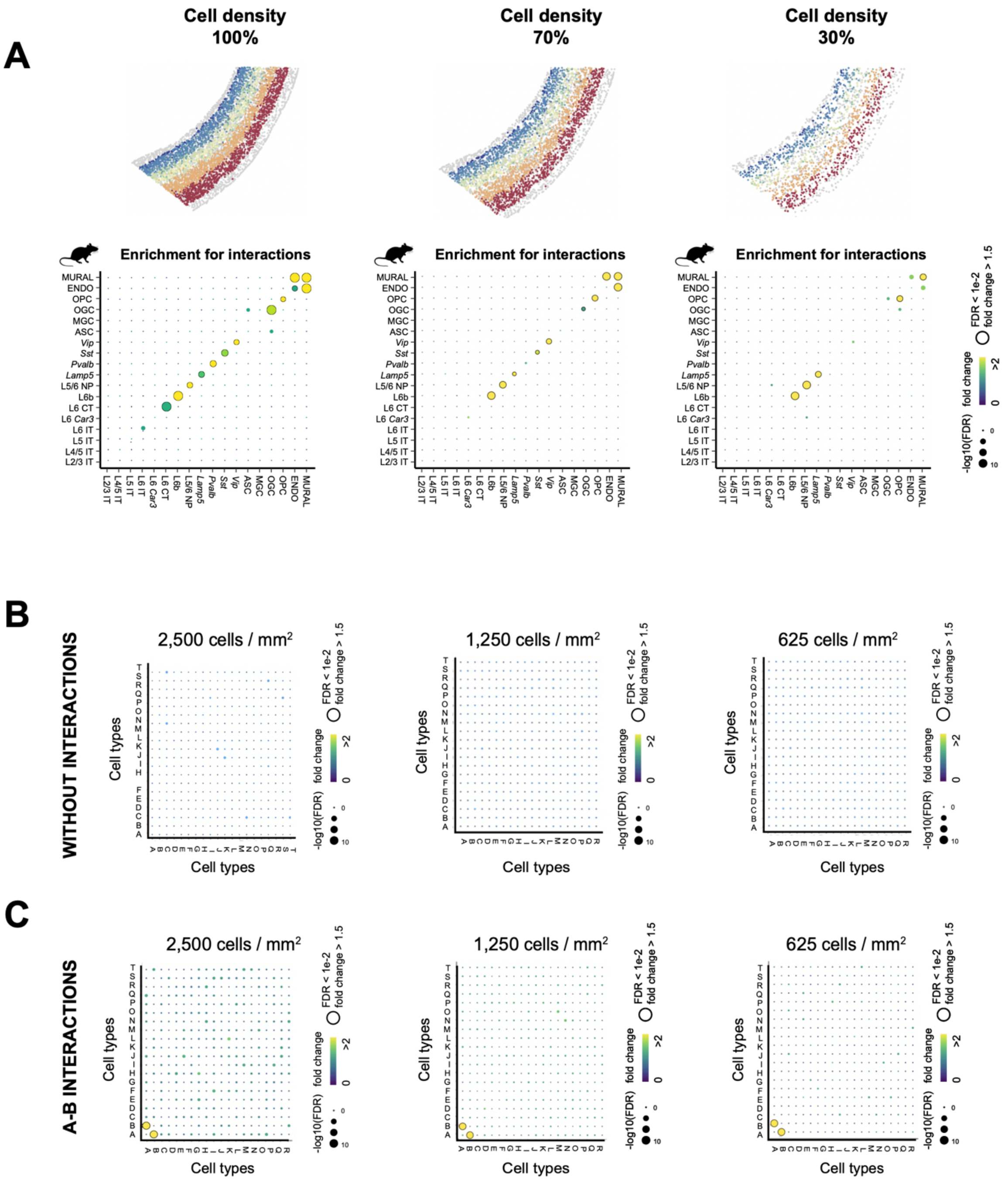

Fig. S15. The effect of cell density on soma contact or proximity enrichment analysis. (**A**) Pairwise soma contact or proximity enrichment map for subclasses of cells in the mouse cortex down-sampled to different cell densities. From left to right: without down-sampling, with down-sampling to 70% and 30% of the original density. The latter case has a similar cell density to the human cortex. (**B**) Pairwise soma contact or proximity enrichment map for simulated data without cell-cell interactions at

different cell densities. The simulation includes 20 cell types, approximately the number of observed subclasses of cells in the human and mouse cortex. We simulated cells with random spatial distributions (no cell-cell interactions) at densities in the range of 625 - 2,500 cells / mm^2, the lower and upper bounds of which are similar to the cell densities in the human and mouse cortex, respectively. (**C**) As in (**B**) but with cell-cell interactions imposed between two cell types A and B: 5% of cells from cell type A interacting with 5% of cells from cell type B, while the remaining cells are randomly distributed.

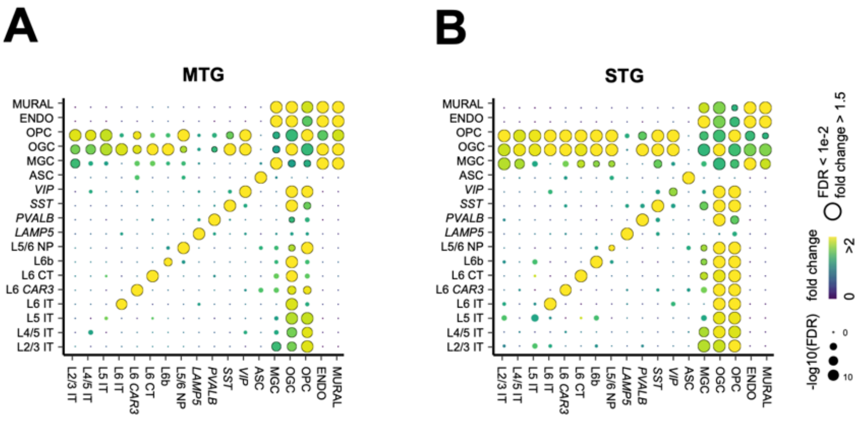

Fig. S16. Pairwise soma contact or proximity enrichment map for subclasses of cells in the human MTG and STG. (**A**) MTG. (**B**) STG. The color and size of the dot are as describe in **Fig. 4B**.

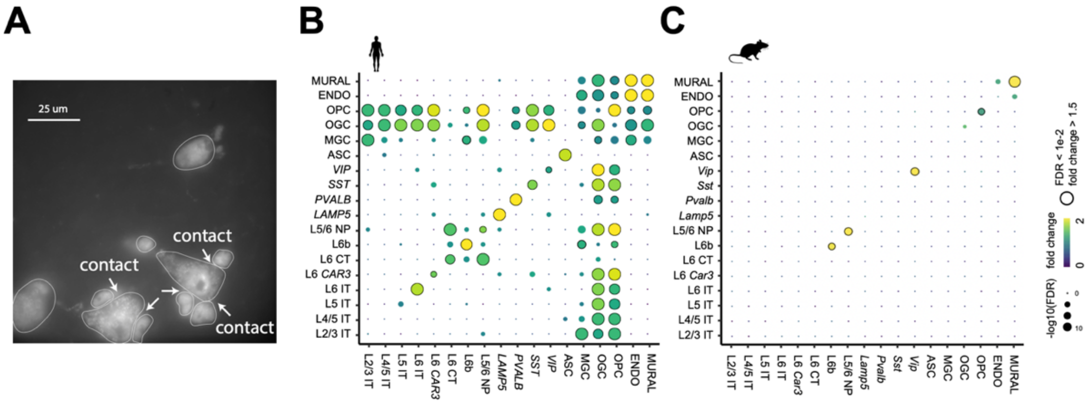

Fig. S17. Pairwise cell-cell contact enrichment map determined using segmented cell boundaries. (**A**) Schematic for soma contact determination using segmented cell boundaries. (**B**) Pairwise soma contact enrichment map for subclasses of cells in the human cortex. (**C**) Pairwise soma contact enrichment map for subclasses of cells in the mouse cortex. The color of the dots indicates the fold change between the observed frequency of soma contact and the average expected frequency from the spatial permutations that disrupt the spatial relationship between neighboring cells (**fig. S13**). The size of the dots indicates the significance level of the enrichment. FDR: P-value determined with upper-tailed Z-test and adjusted to FDR by the BH procedure.

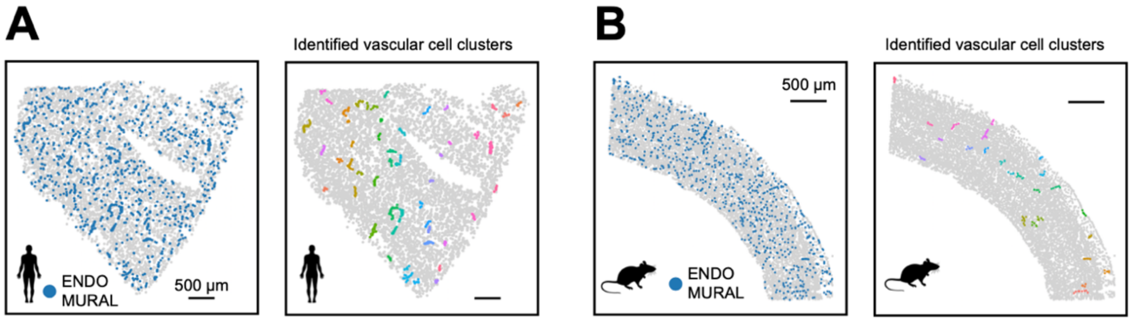

Fig. S18. Identification of vascular structures. (**A**) (Left) Spatial map of vascular cells in a human MTG slice. (Right) Identified clusters of vascular cells in the slice. Line-shaped vascular structures, presumably blood vessels, were identified based on the density of the vascular cells and shown in colors. A k-nearest neighbor (KNN) graph was created between vascular cells in which each node represents a vascular cell, and an edge was drawn between two nodes if their distance was within 40 µm in the imaging plane. We next identified connected components in the KNN graph and components with cells fewer than six were filtered out. (**B**) As in (**A**) but for a mouse slice that contained the VIS, AUD and TEa.

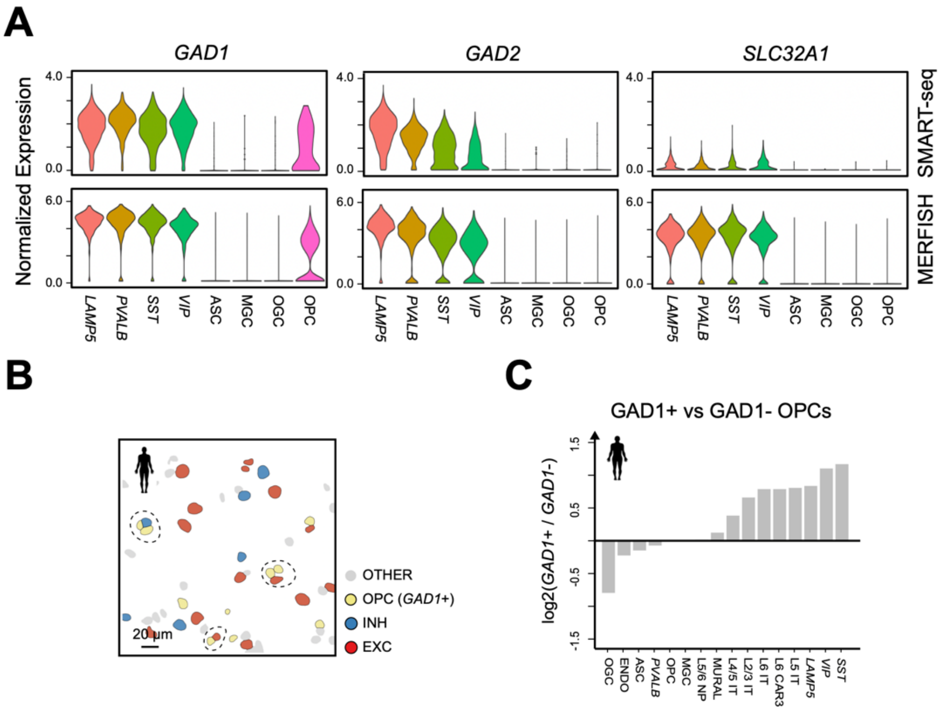

Fig. S19. Different levels of soma interactions between two different subpopulations of OPCs and neurons. (**A**) Normalized expression level of *GAD1, GAD2* and *SLC32A1* in subclasses of inhibitory neurons and glial cells in human MTG and STG determined by SMART-seq (top) and by 4,000-gene MERFISH (bottom). (**B**) Spatial maps of the excitatory neurons, inhibitory neurons, and *GAD1*-positive OPCs in an example region from a human MTG slice. Contacting OPCs and neurons are highlighted by dash line. (**C**) The log2 (fold change) of the contact frequency between *GAD1*-positive OPC and other cell types over the contact frequency between *GAD1*-negative OPC and other cell types.

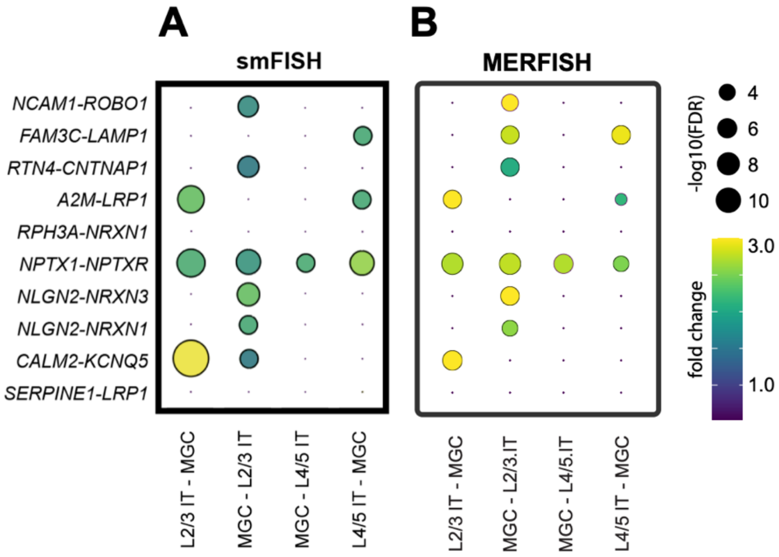

Fig. S20. Enrichment of ligand-receptor pairs in contacting microglia and IT neurons measured by single-molecule FISH (smFISH). (**A**) Enrichment of the ten ligand-receptor pairs shown in **Fig. 5E** in contacting microglia and IT neurons measured by smFISH. The color and size of the dots correspond to the fold change and significance level of the observed ligand-receptor enrichment scores over their expected values. To probe ligand-receptor enrichment in a cell-type specific manner with smFISH, we used *SELPLG* to mark microglia and *SLC17A7* to mark excitatory neurons, and characteristic layer morphology (i.e. L4 has a higher cell density than other layers; L1 has no excitatory neurons) to demarcate L2/3 and L4 approximately. Since in L2/3 and L4, essentially all (>98%) excitatory neurons are IT neurons, we did not use additional marker genes to mark IT neurons. This approach allowed us to identify L2/3 IT and L4/5 IT neurons in the smFISH data. We did not consider L5 and L6 excitatory neurons in the smFISH experiments. (**B**) Corresponding MERFISH results for the ten ligand-receptor pairs in contact microglia and IT neurons. Reproduced from **Fig. 5E** with only results for L2/3 IT and L4/5 IT neurons shown to facilitate comparison with the smFISH results.

Supplementary Tables

Table S1. MERFISH codebook for 4,000-gene measurement. The first column is the gene name, the second column is the Ensembl transcript ID, and the following columns indicate the binary values for each of the 48 bits indicated by name of the corresponding readout sequence. Barcodes that were used as blank controls are denoted by a gene name that begins with “Blank-.”.

Table S2. **MERFISH encoding probe information for 4,000-gene measurement.** For each encoding probe, the encoding probe name and encoding probe sequence are provided. Each encoding probe name also indicates the gene name and Ensembl ID of the targeted transcript, and the names of the associated readout sequences.

Table S3. MERFISH codebook for 250-gene measurement. The first column is the gene name, the second column is the Ensembl transcript ID, and the following columns indicate the binary values for each of the 48 bits indicated by name of the corresponding readout sequence. Barcodes that were used as blank controls are denoted by a gene name that begins with “Blank-.”.

Table S4. MERFISH encoding probe information for 250-gene measurement. For each encoding probe, the encoding probe name and encoding probe sequence are provided. Each encoding probe name also indicates the gene name and Ensembl ID of the targeted transcript, and the names of the associated readout sequences.

Table S5. Readout probe information. For each of the bits, the bit number, the readout probe sequence name, the readout probe sequence is indicated.

Table S6. smFISH probe information. For smFISH probe, the probe name and probe sequence are provided.
